# Effects of Dopants on the Structural, Electronic, and Energetic Properties of (ZrO_2_)_16_ Clusters

**DOI:** 10.1021/acsomega.4c10718

**Published:** 2025-01-29

**Authors:** Priscilla Felício-Sousa, Karla F. Andriani, Marcos G. Quiles, Juarez L. F. Da Silva

**Affiliations:** † São Carlos Institute of Chemistry, University of São Paulo, P.O. Box 780, 13560-970 São Carlos, São Paulo, Brazil; ‡ Departament of Exact Sciences, State University of Santa Cruz, 45662-900 Ilhéus, Bahia, Brazil; § Institute of Science and Technology, Federal University of São Paulo, 12231-280 São José dos Campos, São Paulo, Brazil

## Abstract

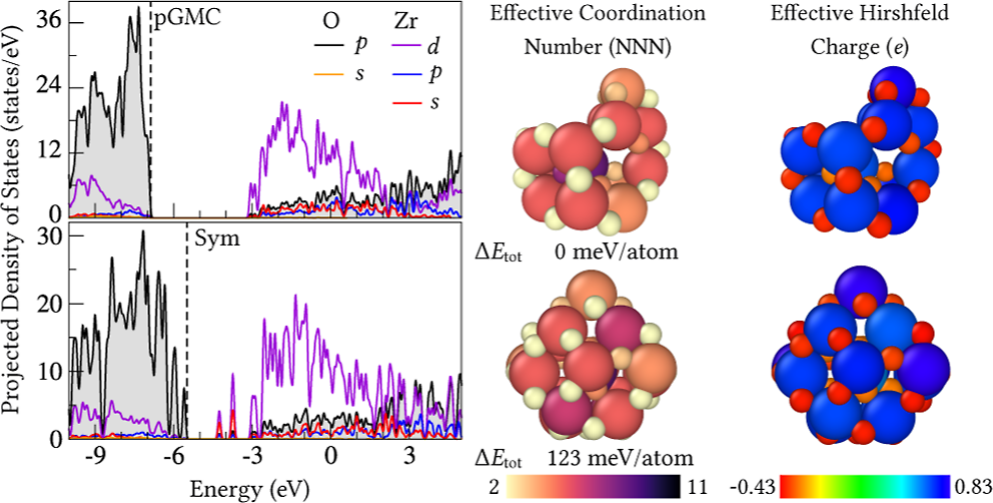

The integration of dopants into ZrO_2_-based clusters provides the possibility to modulate their physicochemical properties, making small clusters promising candidates for various applications, such as catalysis. However, the synergistic interactions between doping and adsorption of single atoms into ZrO_2_ remain poorly understood. Therefore, in this study, we investigate the influence of lanthanum (La) doping and rhodium (Rh) single-atom adsorption on the physicochemical properties of (ZrO_2_)_16_ clusters using density functional theory calculations combined with data science approaches. We found that both doping and adsorption processes lead to minor local structural changes. La doping induces minimal distortions while preserving the overall stability of the cluster, as evidenced by consistent binding energy values. Rh adsorption has a preference to bind near the O–La moieties. In contrast, the electronic structure is majorly affected by Rh adsorption, by narrowing the HOMO–LUMO energy gap, and enhancing the reactivity of those modified Zr_16_O_32_ clusters. Furthermore, Hirshfeld charge analysis reveals a significant charge redistribution following La doping, which is enhanced by the adsorption of a single Rh atom, resulting in localized electronic changes.

## 1 Introduction

The integration of few atoms or even single-atom dopants into oxide-based substrates, referred here as single-atom catalysts (SACs), represents a promising strategy for the development of a novel class of catalytic systems.^1−4^ For instance, the examination of different single atoms supported on the same substrate exhibits distinct reactivity patterns, e.g. PtAl_2_O_4_^–^ produces formaldehyde while RhAl_2_O_4_^–^ converts methane to syngas.^5,6^ The superior performance exhibited by these systems compared to traditional catalysts can be attributed to the unique electronic properties arising from the synergistic interactions between the SACs and the support, distinguishing them from conventional bulk and nanoparticle catalysts.

Several studies have shown that noble-metal SACs, such as Rh/CeO_2_–ZrO_2_, Ni/CeO_2_, and Co/CeO_2_, can enhance the catalytic performance, in particular for methane activation.^7,8^ Furthermore, Rh/ZrO_2_ can selectively produce methanol or ethane from methane, avoiding the overoxidation products like carbon dioxide typically observed with Rh nanoparticles.^9^ Similarly, single-atom cobalt dopants supported on ZrO_2_ have shown excellent efficiency in CO_2_ fixation, achieving a near-optimal yield of carbonate products.^10^

Among various oxide catalysts, zirconia oxide (ZrO_2_) has been identified as a highly adaptable and potent catalyst for methane conversion and alkane activation. Its catalytic efficacy has been attributed to the presence of Zr^4+^ cations with lower coordination located near the oxygen vacancies, which facilitate dissociation of the C–H bond.^11^ By modulating the size of the crystallite and incorporating dopants, the concentration of these active sites can be modified, thus enhancing catalytic performance, particularly in the context of nonoxidative dehydrogenation of alkanes.^12^ Moreover, zirconia-ceria mixed oxides have been employed to facilitate the oxidative transformation of methane into higher hydrocarbons, thereby demonstrating superior redox characteristics and enhanced oxygen mobility.^13^ Sulfated ZrO_2_ similarly shows substantial reactivity in the activation of light alkanes, with its catalytic efficiency being dependent on the oxidation of alkanes to alkenes followed by the formation of carbenium ions.^14^

As demonstrated by Zhao et al.,^15^ Rh doping on ZrO_2_ surfaces enables the selective oxidation of methane to methanol, with four-coordinated Rh sites playing a crucial role in stabilizing the CH_3_ intermediate and preventing overoxidation.^15,16^ Similarly, the work of Okolie et al.^17^ shows that nickel oxide clusters supported on ceria-zirconia are promising for methane conversion to methanol and ethanol under moderate conditions, using O_2_ as an oxidant. This enhanced performance is attributed to its distinct electronic and geometric features, as well as the formation of stable surface hydroxyl groups, which favors the production of methanol.^18^ For the partial oxidation of methane to syngas, single Rh_1_O_5_ clusters on TiO_2_ achieve 97% selectivity and high durability at 650 °C through high-temperature catalysis.^19^

These findings highlight the versatility and effectiveness of catalysts based on ZrO_2_ in various alkane conversion processes, underscoring their potential for industrial applications. However, our understanding of the physicochemical descriptors that govern the structural and electronic properties of SACs remains limited, particularly in the context of ZrO_2_-based clusters. Thus, improving our atomistic understanding of single-atom defects in ZrO_2_ clusters can play an important role due to their transformative potential in catalysis. These defects, formed by integrating single-atom dopants into oxide surfaces combined with quantum-size effects, enable unique electronic properties. Thus, in this study, we employed density functional theory (DFT) calculations combined with data science approaches to investigate the effects of including La dopants and Rh single atoms in the Zr_16_O_32_ cluster models on their structural, energetic and electronic properties.

## 2 Theoretical Approach and Computational Details

### 2.1 Total Energy Calculations

All calculations were based on the spin-polarized DFT^20,21^ framework employing the semilocal formulation proposed by Perdew–Burke–Ernzerhof (PBE)^22^ for the exchange–correlation energy functional. To improve the accuracy of the PBE functional, we used the van der Waals (vdW) correction proposed by Tkatchenko–Scheffler (TS),^23^ which adds an attractive vdW energy correction to the plain DFT-PBE total energy, expressed as *E*_tot_ = *E*_tot_^DFT–PBE^ + *E*_energy_^vdW^.

To solve the Kohn–Sham (KS) equations, we used the all-electron Fritz–Haber Institute the ab initio materials simulation package (FHI-aims),^24,25^ where the KS orbitals are expanded into numerical atom-centered orbitals (NAOs).^24,26^ These NAOs are hierarchically assembled from the minimal basis set free atom orbitals up to the second improvement of the basis set, known as *light-tier2* (following the terminology provided by FHI-aims).^24,25^ The electrons were treated using the scalar relativistic framework within the atomic zeroth order relativistic approximation (atomic ZORA).^27^

The self-consistent charge density was obtained once the total energy, atomic forces, electron density, and sum of the eigenvalues met the convergence criteria of less than 1.0 × 10^–5^ eV, 1.0 × 10^–3^ eV Å^–1^, 1.0 × 10^–4^*e*, and 1.0 × 10^–2^ eV respectively. A Gaussian broadening parameter of 10 meV was applied to ensure the correct occupation of the electronic states closer to the highest occupied molecular orbitals (HOMO). The modified Broyden–Fletcher–Goldfarb–Shanno (BFGS) algorithm^28,29^ was used to optimize the atomic forces on each atom, and the equilibrium geometry was obtained once the atomic forces on every atom were smaller than 1.0 × 10^–2^ eV Å^–1^. For vibrational frequency calculations, we used the finite difference algorithm to calculate the Hessian matrix. Atomic displacements were established at 1.0 × 10^–3^ Å, with stricter criteria applied to total energy (1.0 × 10^–6^ eV Å^–1^) and atomic forces (1.0 × 10^–5^ eV Å^–1^).

### 2.2 Zirconia-Based Cluster Models

In principle, clusters of any size could be selected to produce a wide range of chemical environments for La substitutional doping or for Rh adsorption. Here, we used several criteria to select the (ZrO_2_)_16_ cluster as the reference system for our studies: (i) available structure models^30^ contain bulk-like features in the core region, (ii) it has been used in multiple theoretical studies,^30−34^ and (iii) it has a relatively small number of atoms (48 atoms), specifically 16 Zr and 32 O atoms, ensuring low computational cost while allowing for the exploration of various molecular models with different chemical environments, particularly for stoichiometric and nonmagnetic structures.^35,36^

#### 2.2.1 Undoped (ZrO_2_)_16_ Clusters

**Figure 1:**
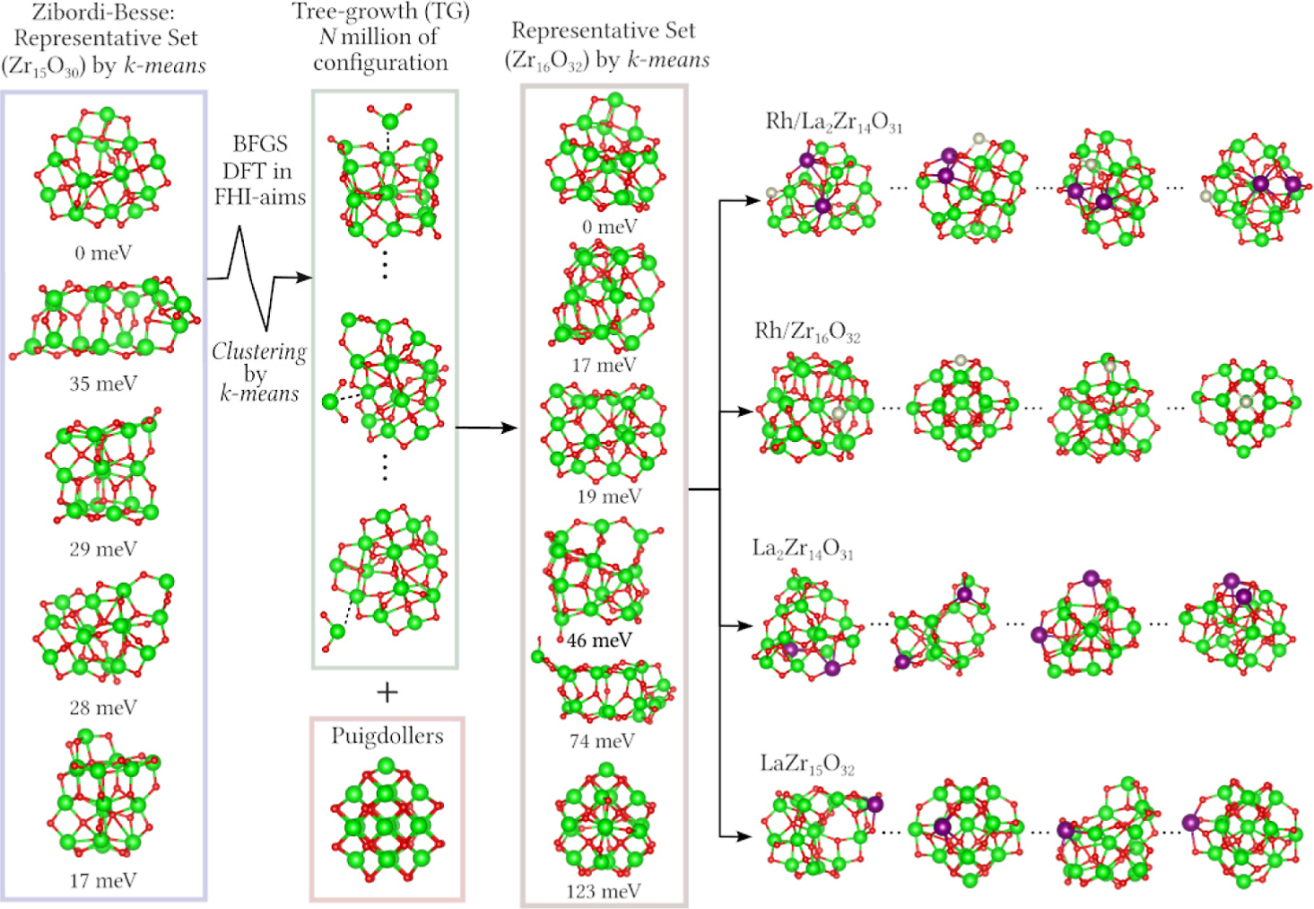
Schematic molecular representation of the design principles used to generate the (ZrO_2_)_16_ clusters from (ZrO_2_)_15_ clusters via tree-growth approach, substitutional doping of Zr by La-atoms, and the adsorption of Rh onto the ZrO_2_-based substrates. The pGMC and the Sym structure were used for doping with 1 or 2 La atoms (purple) and Rh adsorption (gray). Relative total energies are given in meV/atom.

Previously, our group performed a systematic theoretical investigation using DFT-PBE calculations to explore the evolution of the physical-chemical properties of stoichiometric oxide clusters, namely, (MO_2_)_*n*_, where M = Ti, Zr, and Ce and *n* = 1, 2, ..., 14, 15.^37^ In that study, the molecular (MO_2_)_*n*_ configurations were generated by a tree-growth (TG) approach^38^ using fragment formula units (MO_2_) combined with the Euclidean similarity distance (ESD) algorithm^39,40^ to reduce the number of configurations. Here, we used an improved strategy to obtain structural models for the (ZrO_2_)_16_ clusters, see Figure 1. It is also based on the TG approach starting from the reoptimized (ZrO_2_)_15_ cluster.^37^ However, we use the *k*-means clustering algorithm^41^ combined with the Coulomb matrix representation^42^ to group molecular structures^43^ instead of the EDS algorithm,^39,40^ because we also incorporated energetic and electronic properties into the *k*-means clustering process.

For example, we selected five representative (ZrO_2_)_15_ clusters from the Zibordi-Besse study,^37^ that also include the putative global minimum configuration (pGMC) for the (ZrO_2_)_15_ cluster. For each of the selected (ZrO_2_)_15_ clusters, we generated millions of configurations for the adsorption of a single ZrO_2_ fragment. Specifically, the formula unit fragment was randomly placed approximately 1.5 Å above the clusters. Therefore, to reduce the number of configurations, we applied the *k*-means clustering algorithm, which reduced the initial configurations from millions to around 20 representative configurations for each cluster. This procedure generated approximately 100 initial trial configurations for ZrO_2_/(ZrO_2_)_15_.

Furthermore, we incorporated symmetric structures derived from bulk fragments into the data set, including the model structure proposed by Puigdollers.^44^ The Puigdollers structure was chosen for its high symmetry (called hereinafter Sym), as it was obtained from the tetragonal structure (t-ZrO_2_). Finally, the DFT-PBE + vdW framework was used to optimize all proposed model configurations for the (ZrO_2_)_16_ cluster. This step was crucial in generating clusters with diverse chemical environments, facilitating the introduction of La substitutional doping and single Rh-atom catalysts.

#### 2.2.2 Lanthanum Substitutional Doping of (ZrO_2_)_16_ Clusters

We designed the following model structures for substitutional La doping in (ZrO_2_)_16_: (i) single substitution of Zr by a La atom (LaZr_15_O_32_), and (ii) double substitution of Zr atoms by two La atoms accompanied by the removal of one O atom to satisfy the electron counting rule, resulting in La_2_Zr_14_O_31_, that is, to minimize the occurrence of unpaired electrons. To generate the first structures, we explore all 16 possible Zr positions within the (ZrO_2_)_16_ cluster. Meanwhile, for the double substitution case, we applied the *k*-means algorithm to select 32 structures for each initial configuration, resulting in a total of 64 configurations for optimization.

#### 2.2.3 Single-Atom Rh Supported in the (ZrO_2_)_16_ Clusters

Beyond substitutional doping using La^3+^ ions, we integrate Rh atoms into our models to investigate their effects on the electronic and structural properties. To obtain structural models, we specifically selected four clusters, two undoped (ZrO_2_)_16_ clusters and two clusters with substitutional La doping. Within each cluster, the Rh atom was positioned at random adsorption sites on the surface, yielding millions of potential configurations. Subsequently, the *k*-means clustering algorithm was used to condense these possibilities into 32 representative configurations per cluster for further optimization.

## 3 Results and Discussion

### 3.1 Relative Total Energies

**Figure 2:**
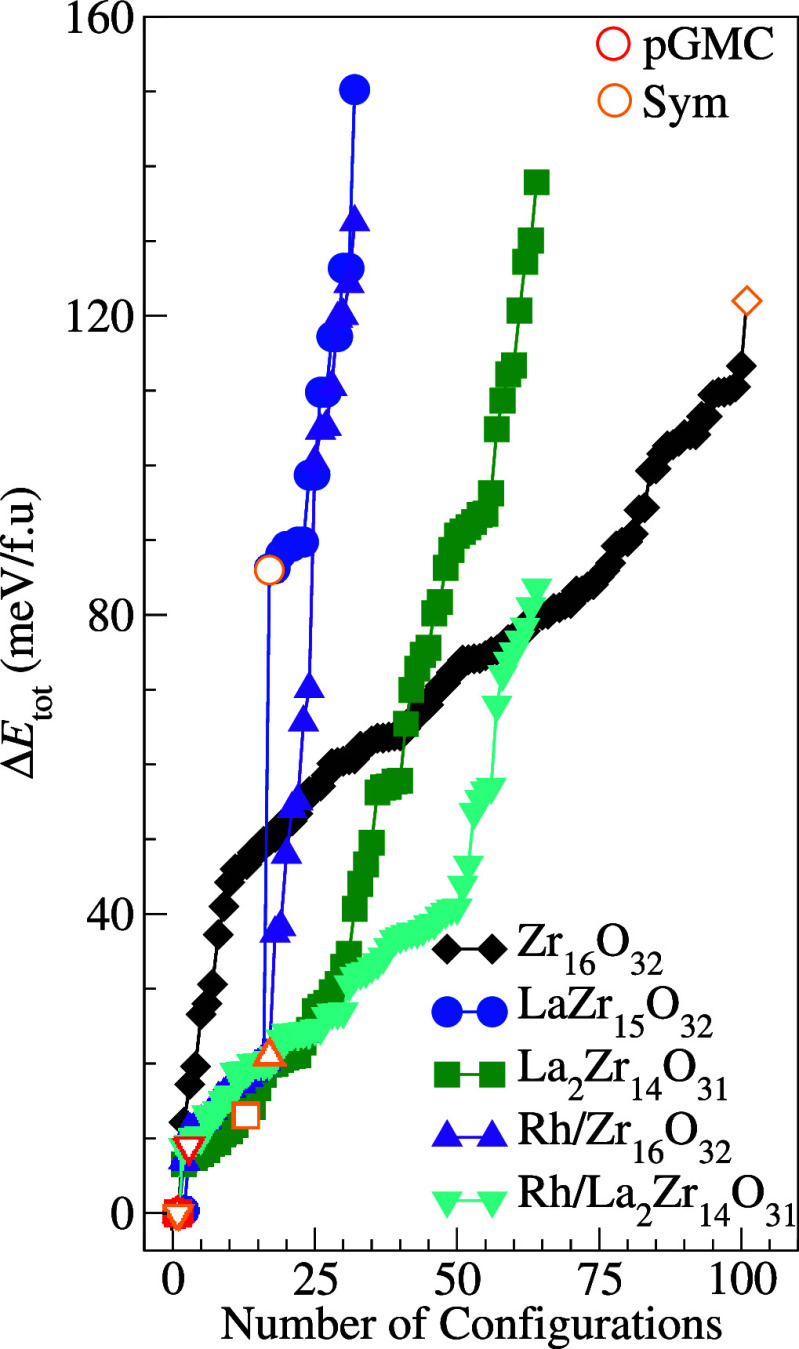
Relative total energies (Δ*E*_tot_) for all optimized ZrO_2_-based clusters, where Δ*E*_tot_ = *E*_tot_^*i*^ – *E*_tot_^lowest^. The pGMC and Sym labels indicate indicate putative global minimum configurations and Puigdoller’s high-symmetry structures, respectively, or the ZrO_2_-based clusters derived from that.

Using the framework described above, we obtained a large set of optimized structures for the Zr_16_O_32_, LaZr_15_O_32_, La_2_Zr_14_O_31_, Rh/Zr_16_O_32_, and Rh/La_2_Zr_14_O_31_ systems. Each optimized structure *i* has a corresponding total energy (*E*_tot_^*i*^), and analyzing the statistical distribution of these energy values provides information on the quality of the initial configurations, as well as the dependence of *E*_tot_^*i*^ on the adsorption sites, which plays a crucial role in the nature of molecule–cluster interactions. The relative total energies (Δ*E*_tot_ = *E*_tot_^*i*^ – *E*_tot_^lowest^) are shown in Figure 2 for all optimized configurations.

For the undoped Zr_16_O_32_ cluster, all optimized ZrO_2_/Zr_15_O_30_ configurations have lower energy than the high-symmetry Puigdoller structure.^45^ Therefore, this result highlights a strong preference for low-symmetry Zr_16_O_32_ structures over high-symmetry alternatives for small oxide clusters. Although several previous DFT studies have employed the Puigdoller model for DFT simulations,^33,34,44^ to our knowledge, this is the first time such findings have been reported in the literature. The Sym structure has an energy of 123 meV/atom higher than the pGMC structure. Therefore, this energy difference suggests that the high-symmetry structure may exhibit different reactivity patterns upon adsorption of molecular species compared to the low-symmetry pGMC structure.

Therefore, given the energy differences and unique geometric characteristics, both the pGMC and Sym structures were selected as templates for substitutional doping of La and the incorporation of single Rh atom catalysts in clusters based on ZrO_2_. As shown in Figure 2, the substitutional doping or single Rh-atom catalyst reduce the energy difference between the structures of pGMC and Sym Zr_16_O_32_. Furthermore, this energy reduction upon modification can stabilize the initial higher energy configurations, which further increases the use of ZrO_2_-based materials for adsorption and catalytic applications.

### 3.2 Selected ZrO_2_-Based Clusters

**Figure 3:**
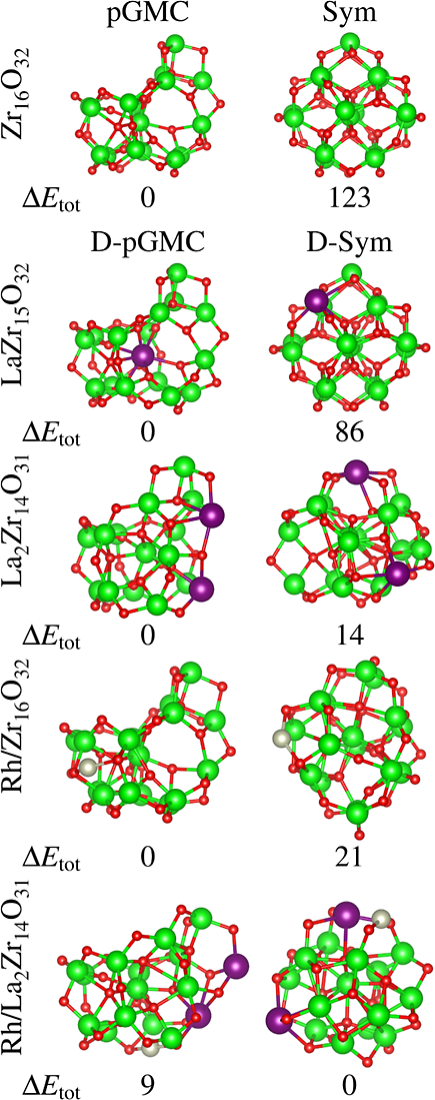
Molecular representation of selected ZrO_2_-based clusters: (a) undoped pGMC and Sym cluster, (b) La substitutional doping, and (c) single Rh-atom supported on clusters. The relative total energies between both structures (Δ*E*_tot_, in meV/atom) are reported below the clusters.

We selected a set of 10 optimized ZrO_2_-based clusters, each representing distinct chemical environments, for example, different species (Zr, O, La, and Rh) exposed directly to the vacuum region. These ten clusters are depicted in Figure 3 along with their relative total energies (Δ*E*_tot_). In addition, the key physical-chemical properties relevant for adsorption investigations are presented in Table 1. Further insights are provided through the project density of states (pDOS) and vibrational analysis depicted in Figure 7, effective coordination number (ECN) calculations in Figure 5 and the Hirshfeld charge distribution in Figure 8.

**Table 1:** Energetic, Electronic, and Structural Properties of the Zr_16_O_32_, LaZr_15_O_32_, La_2_Zr_14_O_31_, Rh/Zr_16_O_32_, and Rh/La_2_Zr_14_O_31_ Clusters: Binding Energy per Atom (*E*_b_), Total Magnetic Moment (*m*_tot_), HOMO Energy (ϵ_H_), LUMO Energy (ϵ_L_), LUMO–HOMO Energy Band Gap (*E*_g_), Average Bond Length for M = Zr, La, and Rh (*d*_av_^M^), Average Bond Length for O Atoms (*d*_av_^O^), and Average Cluster Radius (*R*_a*v*_)

cluster	model	E_b_ (eV/atom)	*m*_tot_ (μ_B_)	ϵ_H_ (eV)	ϵ_L_ (eV)	*E*_g_ (eV)	*d*_av_^M^ (Å)	*d*_av_^O^ (Å)	*R*_av_ (Å)
Zr_16_O_32_	pGMC	–7.25	0	–6.88	–2.95	3.93	2.06	2.06	5.70
	Sym	–7.13	0	–5.50	–4.16	1.33	2.10	2.10	4.70
LaZr_15_O_32_	D-pGMC	–7.24	1	–6.78	–5.77	1.01	2.26	2.07	5.66
	D-Sym	–7.13	1	–5.93	–5.60	0.32	2.18	2.12	4.77
La_2_Zr_14_O_31_	D-pGMC	–7.10	0	–6.53	–2.47	4.05	2.19	2.08	5.78
	D-Sym	–7.11	0	–6.14	–2.71	3.44	2.20	2.08	4.97
Rh/Zr_16_O_32_	D-pGMC	–7.32	1	–4.70	–3.90	0.80	2.05	2.06	5.56
	D-Sym	–7.30	1	–5.25	–4.14	1.10	2.07	2.07	5.26
Rh/La_2_Zr_14_O_31_	D-pGMC	–7.19	1	–4.37	–3.46	0.91	2.21	2.09	5.77
	D-Sym	–7.18	1	–4.04	–3.42	0.63	2.16	2.07	5.04

### 3.3 Energetic Stability

#### 3.3.1 Binding Energy

To characterize the energetic stability of the selected clusters, we calculated the binding energy per atom (*E*_b_) relative to the free-atoms using the following equation
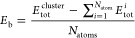
1where *E*_tot_^cluster^ is the total energy of the equilibrium cluster configuration, while *E*_tot_^*i*^ indicates the total energy of the free-atoms, and *N*_atoms_ is the total number of atoms in the cluster. Consequently, as defined, the binding energy per atom quantifies the energy acquired per atom during the formation of a cluster from isolated atoms. Therefore, higher absolute values of *E*_b_ suggest greater stability. The results *E*_b_ are shown in Table 1.

We observed that the magnitude of *E*_b_ for the representative configurations is close to that found of undoped Zr_16_O_32_, with values of −7.25 eV (pGMC) and −7.13 eV (Sym), as previously reported by Zibordi-Besse et al.^37^ Across the representative systems, the *E*_b_ variation remained within a narrow range, between −7.32 and −7.10 eV. Although the ionic radius of La is considerably larger than that of Zr, the structural distortion resulting from La doping was minimal and did not significantly affect the binding energy. In some systems, a slight increase in binding energy was observed, which may be associated with the formation of structural defects, such as oxygen vacancies or interstitials, due to doping.

The proximity of the binding energy values between the doped and undoped clusters suggests that incorporation of La and Rh dopants maintains the overall stability of the system. This insight indicates that the structural and electronic changes induced by doping are balanced by other stabilizing factors, resulting in comparable overall binding energies across the different configurations.

#### 3.3.2 Adsorption Energy

To better understand the strength of interactions between Rh atoms and clusters, we calculated the adsorption energy (*E*_ad_) and the interaction energy (*E*_int_) for Rh adsorbed on the cluster supports. These properties provide valuable information on the energetic stability and nature of the interactions between the Rh and the cluster. *E*_ad_ was calculated using by the following equation
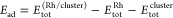
2where *E*_tot_^(Rh/cluster)^ is the total energy of the adsorbed Rh/cluster system, *E*_tot_^Rh^ is the total energy of the free Rh atom, and *E*_tot_^cluster^ is the total energy of the cluster in the gas phase.

Furthermore, *E*_int_ was calculated using the equation
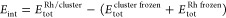
3where, *E*_tot_^cluster frozen^ (cluster = Rh/Zr_16_O_32_ and Rh/La_2_Zr_14_O_31_) and *E*_tot_^Rh frozen^ are, in the frozen geometry of the adsorbed system, the total energies of the cluster and the free Rh atom, respectively. We calculated *E*_ad_ and *E*_int_ for Rh/Zr_16_O_32_ and Rh/La_2_Zr_14_O_31_ using the D-pGMC and D-Sym clusters, where the prefix D- signifies structures that have been derived from the pGMC and Sym configurations.

**Figure 4:**
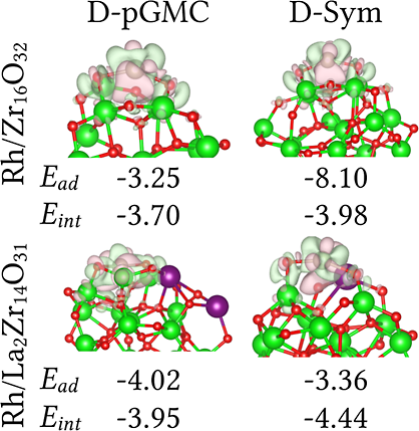
Rh single-atom on (Rh/Zr_16_O_32_ and Rh/La_2_Zr_14_O_31_), D-pGMC and D-Sym clusters. Electron density difference Δρ in eÅ^–3^, adsorption energy (*E*_ad_ in eV), and interaction energy (*E*_int_ in eV), where Zr (green), La (purple), Rh (silver), O (red). The light green (light red) isosurfaces (0.015 eÅ^–3^ cutoff) indicate accumulation (depletion) of the charge.

Our results show that the negative values of the adsorption energy indicate that the adsorption of the Rh atom onto the Zr_16_O_32_ cluster is energetically favorable for all representative configurations, as shown in Figure 4. Furthermore, the higher magnitude of *E*_ad_ in the D-Sym structure (−8.10 eV) indicates a stronger bond between Rh atom and the cluster surface compared to the D-pGMC cluster (−3.25 eV). However, the lower magnitude of the interaction energy in the D-Sym structure (−3.98 eV) compared to *E*_ad_ suggests an unfavorable contribution of the deformation energy due to geometrical changes in the Zr_16_O_32_ cluster from its gas phase geometry to accommodate the Rh atom. In contrast, the deformation energy is a stabilizing factor for the D-pGMC cluster, with *E*_int_ = −3.70 eV.

In the Rh/La_2_Zr_14_O_31_ system, the D-pGMC cluster (−4.02 eV) exhibits slightly stronger bonding compared to the D-Sym cluster (−3.36 eV). These negative values further indicate that the Rh atom is energetically stable upon adsorption onto the La_2_Zr_14_O_31_ cluster. The relatively higher adsorption energy magnitudes in the Rh/La_2_Zr_14_O_31_ systems suggest a stronger bonding between the Rh atom and the La_2_Zr_14_O_31_ cluster compared to the Rh/Zr_16_O_32_ system. In these systems, the deformation energy acts as a mild destabilizing factor in the D-pGMC cluster (*E*_int_ = −3.95 eV) but serves as a stronger stabilizing factor in the D-Sym cluster (*E*_int_ = −4.44 eV), suggesting that the deformation of the cluster geometry stabilizes the Rh atom.

### 3.4 Analysis of the Structural Features

We selected several descriptors for structural analysis, including the average effective coordination number (ECN_av_),^46,47^ average weighted bond length (*d*_av_),^48^ chemical order parameter (σ),^49,50^ average particle radius (*R*_av_), identification of the chemical species exposed to the vacuum region. The results are summarized in Table 1 and Figure 3, while their mathematical formulation is reported in the Supporting Information file.

#### 3.4.1 Cluster Radius

The results show a wide variation in cluster radius size, ranging approximately from 4.0 to 9.0 Å. This variation arises from the use of five distinct structures for (ZrO_2_)_16_ growth. Therefore, the elongated configurations can be attributed to the inclusion of a structure that, during its formation process, exhibited characteristics of alternating layers of zirconia and oxygen, i.e., it resembles a bilayer ZrO_2_ structure.^51^ Consequently, as growth proceeded, the bonding of monomers at the ends of this structure generated more elongated configurations.

Furthermore, the structures used in doping had an average radius of 5.7 to 4.7 Å for D-pGMC and D-Sym, respectively, Table 1. In the latter, the smallest variation in *R*_av_ occurs because the Sym configuration preserves the symmetry derived from the t-ZrO_2_ phase. Thus, in configurations with substitutional and interstitial doping, *R*_av_ ranged from 4.0 to 6.0 Å due to the limited use of D-pGMC and D-Sym configurations. However, we observed an increase in *R*_av_ with insertion of dopants, since La (1.95 Å) present a larger cationic radius than Zr (1.55 Å).^52^ Therefore, the insertion of an atom in the interstice causes an expansion of the cluster lattice.

#### 3.4.2 Effective Coordination Number

**Figure 5:**
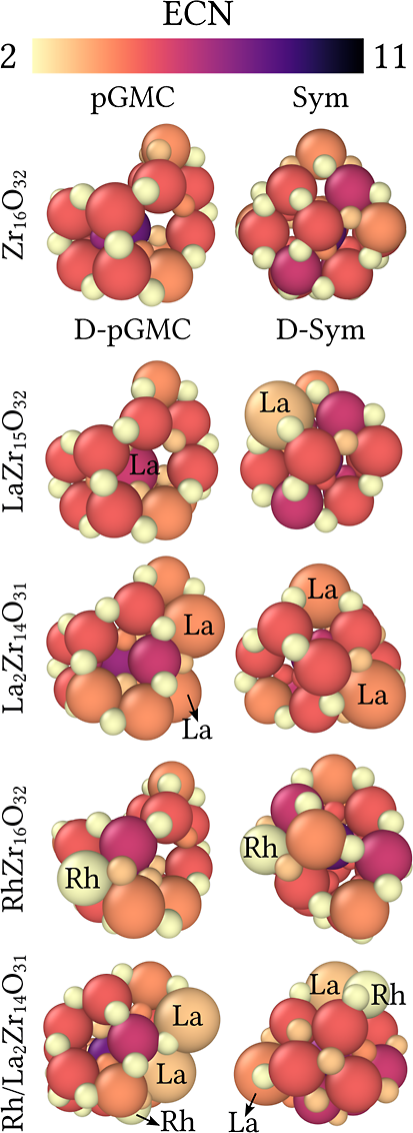
Effective coordination number given in number of nearest neighbors (NNN). The color gradient indicates variation in the coordination, ranging from 2 NNN (beige) to 11 NNN (dark purple).

Commonly, the average effective coordination number (ECN_av_) generally ranged from 2.5 to 4.0 NNN. However, individual atoms, especially those located in the center of the cluster, exhibit higher coordination, as shown in Figure 5. Surface oxygen atoms tend to have low coordination, especially when coordinated with two-central (O_2c_) or three-central (O_3c_) atoms, making them more easily removable and important as catalytic sites in redox reactions involving oxygen transfer.^53^ Additionally, some zirconium cations display unsaturated coordination (Zr_cus_, where “cus” denotes unsaturated coordination), serving as active sites for nonoxidative alkane dehydrogenation.^53^ Thus, similar trends are observed on t-ZrO_2_ surfaces, where zirconium exhibits three coordination types: Zr_6c_, Zr_7c_, and Zr_8c_.^54^

In the Rh/cluster configuration, Figure 5, the surface Rh atom coordinates with a surface oxygen atom of the ZrO_2_ cluster and an additional oxygen atom, resulting in Rh–2c–O coordination. In contrast, the Rh–1c–O structure represents a stoichiometric configuration. These findings, along with those of Thang and Pacchioni.,^54^ suggest that the Rh–2c–O structure is the most accurate computational model for tetragonal ZrO_2_-supported systems.

#### 3.4.3 Average Weighted Bond Lengths

The insertion of dopants did not significantly alter the average bond lengths (*d*_av_) of the Zr_16_O_32_ cluster. Thus, in undoped systems, these distances ranged from 1.96 to 2.10 Å , while in doped systems, they ranged between 2.04 and 2.11 Å (see Table 1). Due to the finite size of the studied clusters, structural rearrangements may occur to achieve a more stable configuration, leading to localized effects. For example, in structures containing multiple M elements, the bond distances involving M = Zr, La, and Rh are generally longer than the average *d*_av_^O^ distances due to the rearrangements induced by the dopants. The Rh/Zr_16_O_32_ system was an exception, showing a minimal impact of the dopant on the bond lengths.

When comparing the average coordination number (ECN_av_) and the average bond length (*d*_av_) for each Zr_16_O_32_-based cluster, clusters with lower *d*_av_ and higher ECN_av_ exhibit greater stability, indicating stronger and more stable bonding between zirconium and oxygen atoms. Specifically, the pGMC cluster, with an ECN_av_ of 3.45 NNN and a *d*_av_ of 2.05 Å, demonstrates relatively strong bonding and stability, marking it as the most stable configuration among those studied.

#### 3.4.4 Radial Distribution Function

**Figure 6:**
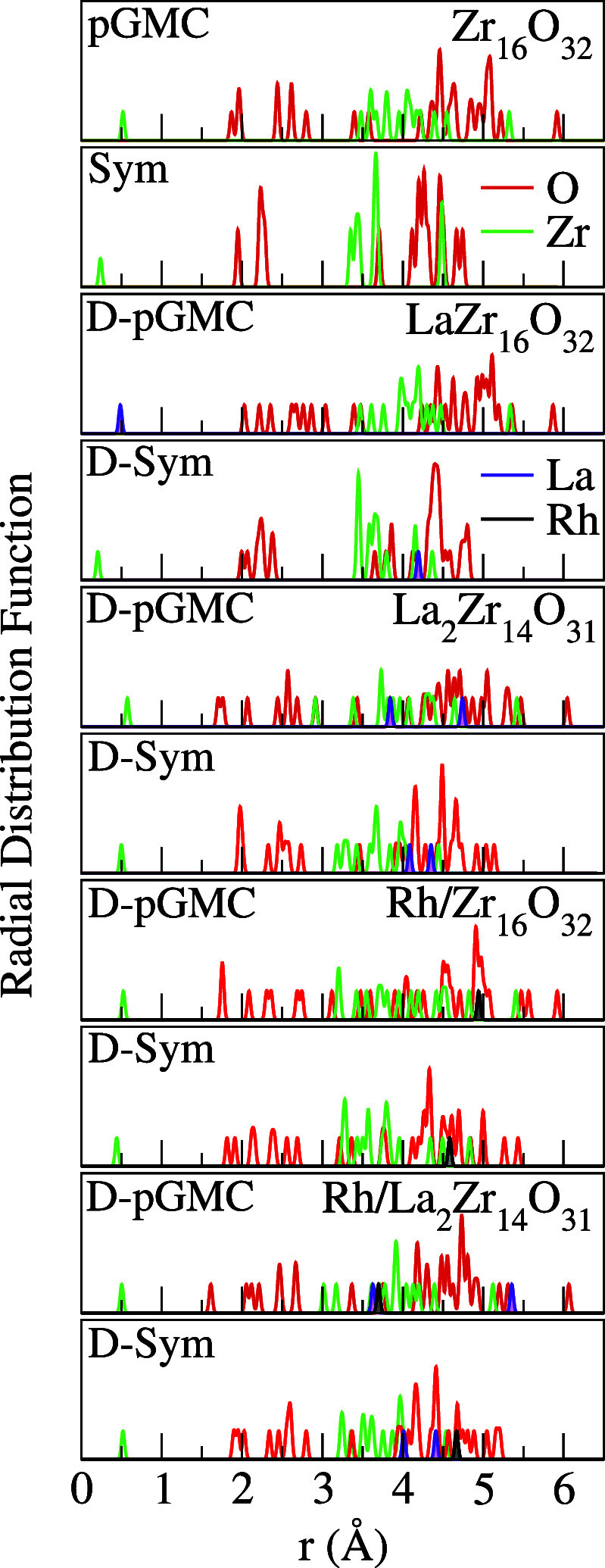
Radial distribution function for all ZrO_2_-based clusters, where solid green, red, purple and black lines represent Zr, O, La and Rh atoms, respectively.

The results in Figure 6 reveal specific trends across all systems: (i) a nearly homogeneous distribution of oxygen atoms throughout the cluster; (ii) the surfaces of the clusters are characterized by the presence of oxygen species in all structures, and (iii) a central core composed of Zr or La atoms in the case of LaZr_16_O_32_ D-pGMC. These findings align with previous results for smaller ZrO_2_ systems.^37^

Furthermore, for the case of Sym structures, typical characteristics of crystalline solids are maintained, with multiple well-defined peaks reflecting periodic ordering. However, this behavior does not occur in structures originating from pGMCs. In addition, D-Sym clusters have a higher number of surface oxygen atoms, a potentially relevant feature for catalytic applications.

Furthermore, only the configuration LaZr_16_O_32_ D-pGMC showed a preference for the La dopant in the center of the cluster, as observed in Figures 3 and 6. This particular configuration induced a symmetry break among the central oxygen atoms compared to Zr_16_O_32_ pGMC, leading to the cluster distortion. However, the distances between the subsequent cations and the oxygen regions within the structure are maintained.

In the Rh/La_2_Zr_14_O_31_ D-pGMC structure, a distancing of the lanthanum cations is observed with the inclusion of Rh. Furthermore, one of the lanthanum atoms migrates to the surface of the cluster, compared to the RDF of La_2_Zr_14_O_31_ D-pGMC. In general, the Rh atoms were adsorbed onto the surface of the cluster, as expected. However, in the single-atom Rh structure, the atoms integrated into the cluster’s Rh/La_2_Zr_14_O_31_ lattice.

### 3.5 Electronic Structure

#### 3.5.1 Projected Density of States

**Figure 7:**
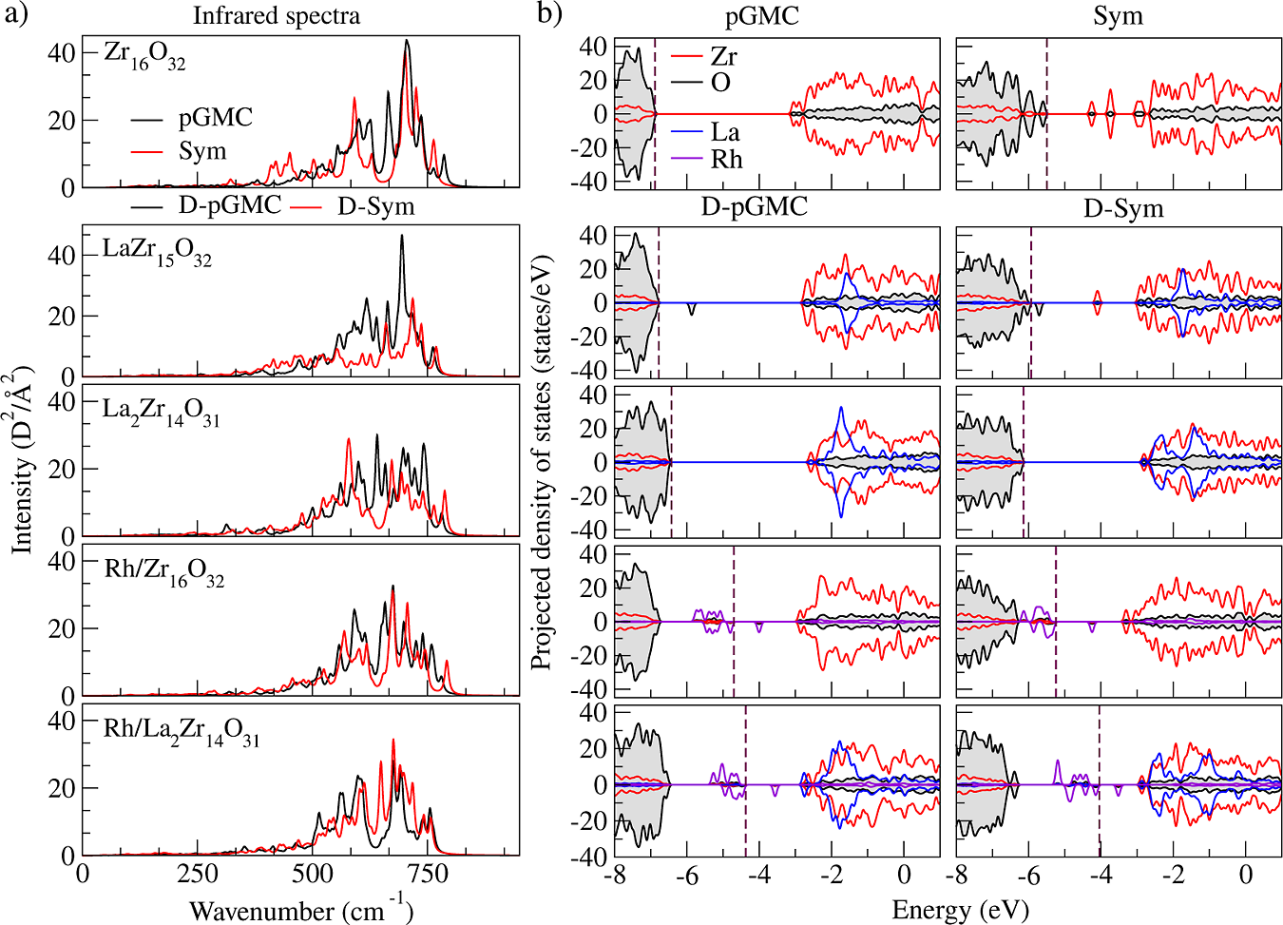
(a) Infrared spectra, where solid black and red lines represent the pGMC and Sym structures, respectively. (b) Projected density of states for all 10 selected ZrO_2_-based clusters. The HOMO energy is indicated by vertical dashed lines, while solid red, black, blue and purple lines, represent the Zr, O, La and Rh states, respectively.

The projected density of states (pDOS) for all 10 selected ZrO_2_-based clusters are shown in Figure 7b. The electronic structure of the zirconia cluster indicates that the valence states are mainly composed of contributions from the O *p*-states, while in the empty states, the largest contribution derives from the Zr *d*-states. Peaks in the valence band within the energy range of −4.5 to 0 eV are primarily due to the *p*-states of oxygen, while those from −4.5 to 1.5 eV arise from the 4 *d*-states of the zirconium atom. A mixing is observed between the O *p*-states and neighboring 4 *d*-states of Zr is observed, suggesting the presence of covalent bonding.

For La-doped clusters, a small localized mixed state appears above the Fermi level, primarily due to the O *p*-states with a minor contribution from the Zr *d*-states. However, when ZrO_2_ is doped with a single Rh atom, localized mixed states emerge above the Fermi level in the energy range between −6 and −4 eV, mainly due to the Rh *p*-states, but also due to the Zr ones, particularly in the D-Sym structure (Rh/La_2_Zr_14_O_31_). Generally, the insertion of substitutionally stable dopants contributes primarily to the conduction states, similar to the undoped ZrO_2_, where the conduction band is predominantly composed of Zr-states. However, this does not apply to structures with a single Rh atom, since the unoccupied states for these structures are derived from the Zr-states with a small contribution from Rh-states.

#### 3.5.2 Analysis of the HOMO and LUMO Energies

Variations in the energies of the HOMO (highest occupied molecular orbital) and LUMO (lowest unoccupied molecular orbital) toward less or more negative values are more pronounced in stoichiometric structures due to orbital occupancy. For example, in the LaZr_15_O_32_ structure, substituting Zr with La results in the formation of lanthanum monoxide (LaO), which is less stable, as reported in the literature,^55^ leading to a smaller magnitude of the HOMO–LUMO energy separation. Consequently, the presence of an additional oxygen may exacerbate this instability.

For Zr_16_O_32_, the pGMC model predicts a band gap of 3.93 eV, consistent with the value reported for the bulk ZrO_2_ in the Baddeleyite phase (∼3.6 eV) at a comparable theoretical level.^37^ In contrast, the Sym model exhibits a reduced band gap of 1.33 eV, indicating a higher reactivity. For example, Puigdollers et al.^45^ calculated the band gap for the Sym (ZrO_2_)_16_ cluster as 1.35 eV and for the bulk ZrO_2_ as 3.75 eV at the PBE level of theory. As expected, the PBE functional underestimates the magnitudes of the band gap.

For example, Gionco et al.^56^ reported a band gap of 5.8 eV for bulk ZrO_2_ using the B3LYP functional, while Puigdollers et al.^45^ observed a value of 5.13 eV with the PBE0 functional. These computational results align more closely with the experimental band gap values, which range from 5.58 to 6.62 eV.^57^ Doping with La in Zr_15_O_32_ increases the HOMO energy and decreases the LUMO energy, resulting in a lower band gap of 1.01 eV in the D-pGMC model and 0.32 eV in the D-Sym model, suggesting increased reactivity and reduced stability compared to Zr_16_O_32_.

In contrast, doping two La atoms in La_2_Zr_14_O_31_ further increases the HOMO energy and decreases the LUMO energy, reflecting greater stability and lower reactivity compared to clusters with fewer dopants (4.05 eV). Furthermore, a single adsorption of Rh atom in Zr_16_O_32_ (3.93 eV) and La_2_Zr_14_O_31_ D-pGMCs (4.05 eV) further increases reactivity by reducing the band gap to 0.80 and 0.91 eV, respectively, thus facilitating the electron transfer process.

### 3.6 Infrared Spectrum

The infrared (IR) spectra for the ten selected oxide clusters are shown in Figure 7, with the following general observations: (i) All selected oxide clusters are confirmed as true local minimum structures, as no imaginary frequencies appear in the IR spectrum; (ii) Thus, the pGMC Zr_16_O_32_ structure serves as an alternative to Puigdoller’s compact structure,^44^ making it suitable for DFT calculations; (iii) Structures with nearby La exhibit characteristic peaks associated with the La–O vibration of La_2_O_3_, in the range of 510–514 cm^–1^.^58^

Furthermore, peaks observed in the range of 640–736 cm^–1^ correspond to the Zr–O vibration. For comparison, the tetragonal phase of zirconia typically shows IR bands within 431–680 cm^–1^ attributed to the same Zr–O vibration mode.^59^ Most peaks in our results appear around 675 cm^–1^, suggesting structural features consistent with t-ZrO_2_. The characteristic experimental peak of Zr–O vibration is reported at 544 cm^–1^.^60,61^ In our results, a similar peak is observed at 576 cm^–1^, which may be shifted due to the incorporation of La in the structure, similar to the findings of La_2_Zr_14_O_3_.

Moreover, La–O bending and stretching modes are observed at 506–518 cm^–1^ and 639–661 cm^–1^, respectively, are consistent in all configurations containing lanthanum. Thus, these values align well with the values in the literature for La_2_O_3_,^58^ where stretching modes appear at 648, 678, and 663 cm^–1^, while bending modes are observed at 510 and 514 cm^–1^, corroborating our findings. However, minor deviations between theoretical and experimental spectra can be attributed to the segregation of La on the surface of the cluster, with only two configurations showing adjacent lanthanum atoms, specifically in La_2_Zr_14_O_32_ and Rh/La_2_Zr_14_O_32_ D-pGMCs. For Rh–O, the bending vibration mode is observed in the range of 543–552 cm^–1^. Experimental data report an IR band centered at 544 cm^–1^ for amorphous rhodium hydrous oxide, heated between 160 and 400 °C, although the specific vibration mode type was not detailed.^62^

### 3.7 Active Sites Based on the Effective Hirshfeld Charges

**Figure 8:**
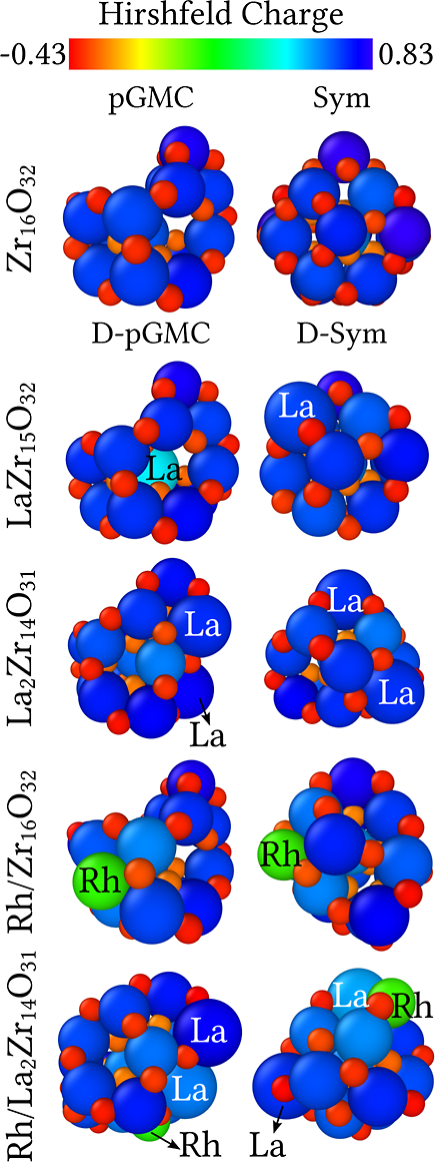
Hirshfeld charge per atom (in e) for the ZrO_2_-based clusters. The color represents the effective Hirshfeld charges on each species, ranging from −0.43 e (red) to 0.83 e (blue).

The Hirshfeld charge analysis provides valuable insight into the effective charges on each atomic site. Hence, it reflects the charge transfer among atomic species using free atoms as reference, which is useful for understanding the mechanisms underlying binding in adsorption process.^63,64^Figure 8 depicts the effective average Hirshfeld charge per atom (Δ*Q*_av_) for Rh (0.11 e), Zr (0.70 e), O (−0.36 e), and La (0.66 e). Furthermore, for the sake of clarity, the predominant oxidation states for Rh and La are 3+, which is more stable, and 4+. In contrast, for Zr, the principal oxidation state is 4+.

The Hirshfeld charge analysis reveals a slight reduction in the positive charge of the Zr cation in La-doped ZrO_2_-based systems, while neighboring O atoms become less negative. This effect is particularly pronounced in the La_2_Zr_14_O_31_ systems. Specifically, in LaZr_15_O_32_ D-pGMC model, the La cation exhibits a lower positive charge (0.49 e), as expected, due to its high coordination with surrounding oxygen atoms. This observation is consistent with previous ECN and RDF analyses.

For the Rh-adsorbed systems, the Rh/Zr_16_O_32_ D-pGMC and D-Sym configurations exhibit distinct effective charges on the Rh cations and neighboring atoms. However, the Rh cations and nearby Zr atoms are less positively charged, consistent with their coordination to surrounding O atoms. In the Rh/La_2_Zr_14_O_31_ systems, one of the La cations shows a notable increase in effective charge, though smaller than that observed for the Rh cations, in both the D-pGMC and D-Sym configurations. Therefore, these clusters present particular characteristics, such as distinct coordinated sites with charge accumulation and depletion, making them potential catalysts for activating molecules such as methane, molecular hydrogen, and others.^63,64^

## 4 Conclusions

In this study, we performed DFT calculations combined with data science algorithms to investigate the effects, at the atomistic level, of La dopants and Rh adsorption on the structural, energetic and electronic properties of ZrO_2_-based clusters. Our results show that the incorporation of La and Rh into the Zr_16_O_32_ cluster slightly affects the structural integrity of the cluster, with only local structural changes observed, which are more pronounced for the adsorption of Rh.

The additions of La and Rh maintain the stability of the system, as evidenced by similar binding energy values among the different structural configurations, that is, high-symmetry and low-energy structures. Furthermore, the La atom generally prefers the surface of the cluster. However, in a specific case, that is, in LaZr_15_O_32_ D-pGMC, the La atom exhibits a preference for the cluster core while remaining exposed to the vacuum due to the features of the structure (cavities). Hence, Rh atoms tend to adsorb near the La doping sites, often binding to O–La moieties.

The insertion of Rh and La significantly changes the electronic properties of the zirconia-based clusters. La doping effects are electronically localized, leading to a decrease of the band gap, i.e., 1.01 eV (pGMC) and 0.32 eV (Sym). Furthermore, Rh adsorption significantly narrows the band gap, i.e., 0.80–0.91 eV, due to the introduction of localized Rh states. Thus, these findings explain the enhanced reactivity of these modified clusters.

Hirshfeld charge analysis reveals significant changes in the effective charge upon La doping and Rh adsorption in (ZrO_2_)-based clusters. As a result, adsorbed Rh exhibits a lower positive charge compared to La, while surrounding Zr atoms display locally reduced positive charges. This behavior indicates partial electron transfer from the cluster, predominantly involving neighboring oxygen atoms, which become less negatively charged relative to the more distant oxygen atoms.

## Data Availability

As mentioned, all DFT calculations were performed using the FHI-aims package,^24,25^ which can be used under a nonfree academic license. Additional details can be obtained from the link, https://aimsclub.fhi-berlin.mpg.de/. Furthermore, the *k*-means clustering algorithm was obtained from https://github.com/quiles/Adsorption_Clus. Additional crude data can be obtained directly from the authors upon request.

## Supporting Information Available

The data used for the figures, as well as complementary analyses and additional technical details, are reported in the Supporting Information (PDF)
