# Supplementary material for: Effects of Dopants on the Structural, Electronic, and Energetic Properties of (ZrO2)16 Clusters
Source: ACS Omega. 2025 Jan 29;10(5):5006–15. doi: 10.1021/acsomega.4c10718 (PMC11822489; doi:10.1021/acsomega.4c10718)
Supplement: Supplementary file 1 — ao4c10718_si_001.pdf [file ao4c10718_si_001.pdf]

Supporting Information File: Effects of Dopants on the Structural, Electronic, and Energetic Properties of $(ZrO_2)_{16}$ Clusters

Priscilla Felício-Sousa,[*,†] Karla F. Andriani,[*,‡,†] Marcos G. Quiles,[*,¶] and Juarez L. F. Da Silva[*,†]

†*São Carlos Institute of Chemistry, University of São Paulo, P.O. Box* 780, 13560-970, *São Carlos, SP, Brazil*

‡*Departament of Exact Sciences, State University of Santa Cruz,* 45662-900, *Ilhéus, BA, Brazil*

¶*Institute of Science and Technology, Federal University of São Paulo,* 12231-280, *São José dos Campos, SP, Brazil*

E-mail: priscillafelicio@me.com; karlandrianiqmc@gmail.com; quiles@unifesp.br; juarez_dasilva@iqsc.usp.br

Contents

S-1 Introduction **S-2**

S-2 Additional Computational Details **S-2**

S-2.1 Self-Consistency Computational Parameters S-2

S-2.2 Computational Convergence Tests S-3

S-2.3 Definition of Selected Physical-Chemical Properties S-4

S-2.4 Representative Cluster Selection S-7

S-3 Additional Data for the Oxide Clusters **S-8**

S-3.1 $Zr_{16}O_{32}$ S-8
S-3.2 $LaZr_{15}O_{32}$ S-11
S-3.3 $La_2Zr_{14}O_{31}$ S-13
S-3.4 Rh/$Zr_{16}O_{32}$ S-15
S-3.5 Rh/$La_2Zr_{14}O_{31}$ S-17

S-4 Atomic Positions of the Lowest Energy Configurations S-20

References S-21

S-1 Introduction

During this study, an extensive series of computations was performed, including several computational convergence tests, optimization of molecular structures, and several analyses. However, due to space constraints, several datasets and analyses could not be included in the main manuscript. Recognizing the importance of supporting data to allow researchers to replicate our findings, we have compiled this document to provide additional details that were omitted from the main manuscript. Furthermore, all the data reported herein were generated using the FHI–aims program package.[1,2]

S-2 Additional Computational Details

S-2.1 Self-Consistency Computational Parameters

In earlier studies, we conducted a series of computational convergence tests,[3] which provided optimal computational parameters required to achieve the self-consistent solution of the Kohn–Sham equation and force optimizations for oxide clusters. For example, 1.0×10^{-5} eV total energy (sc_accuracy_etot), $1.0 \times 10^{-4}\,e$ electron density (sc_accuracy_rho), 1.0×10^{-3} eV Å^{-1} for atomic forces (sc_accuracy_forces), 1.0×10^{-2} eV sum of the eigenvalues (sc_accuracy_eev) and 1.0×10^{-2} eV Å^{-1} for the atomic forces during geometric optimization (relax_geometry trm), respectively.

Therefore, the same set of parameters was used in the present work. Additionally, we performed further tests to evaluate the basis-set accuracy level, as demonstrated in the following section.

Table S-1: Self-consistency computational parameters employed to control the self-consistency process, namely sc_accuracy_etot (total energy), sc_accuracy_rho (electron density), sc_accuracy_eev (sum of the eigenvalues), sc_accuracy_forces (atomic forces) and relax_geometry trm (geometric optimization).

Flags	Values
sc_accuracy_etot	1.0×10^{-5} eV
sc_accuracy_force	1.0×10^{-3} eV Å^{-1}
sc_accuracy_rho	1.0×10^{-4} e
sc_accuracy_eev	1.0×10^{-2} eV
relax_geometry trm	1.0×10^{-2} eV Å^{-1}

S-2.2 Computational Convergence Tests

We performed target tests using several basis sets levels as implemented in FHI–aims, specifically the light-tier and tight-tier levels, to evaluate the basis set accuracy level for the systems under study. For these tests, we used the putative global minimum configurations (pGMC) of the following clusters: $Zr_{16}O_{32}$, $La_2Zr_{14}O_{31}$, Rh/$Zr_{16}O_{32}$ and Rh/$La_2Zr_{14}O_{31}$, Figure S-2. Thus, the precision of the basis set was assessed with respect to the following key parameters: relative total energy (ΔE_{tot}), binding energy (E_b), effective coordination number (ECN) and average bond length (d_{av}). For the ΔE_{tot} tests, the level of the tight-tier3 basis set was used as a reference. The results of these computational convergence tests are summarized in Table S-1.

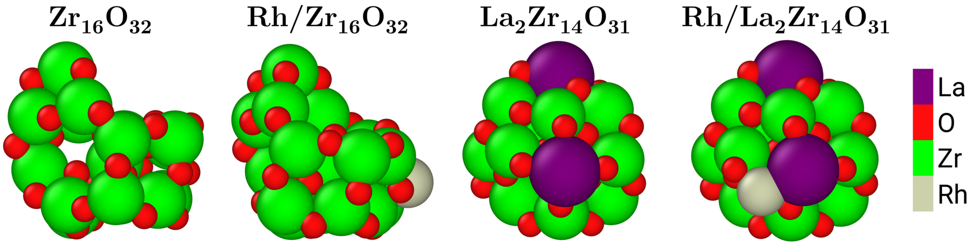

Figure S-1: Selected $Zr_{16}O_{32}$, $LaZr_{15}O_{32}$, $La_2Zr_{14}O_{31}$, Rh/$Zr_{16}O_{32}$ and Rh/$La_2Zr_{14}O_{31}$ oxide clusters for the computational convergence tests.

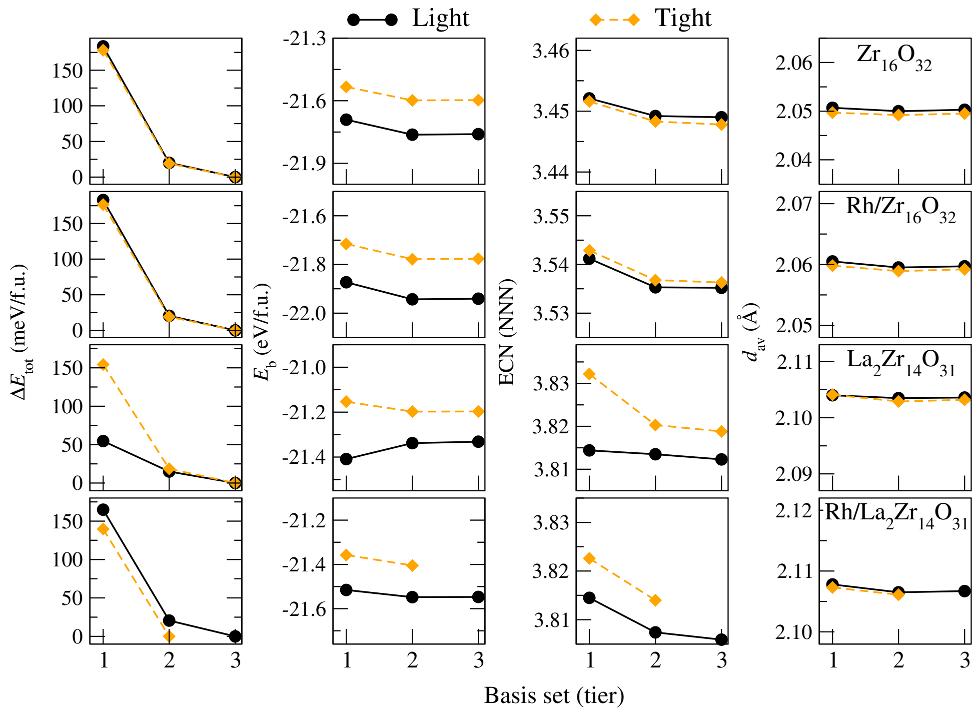

Figure S-2: Computational convergence tests for the pGMC oxide clusters to evaluate the basis set accuracy relative to the following parameters: relative total energy (ΔE_{tot}), binding energy (E_b), effective coordination number (ECN), and average bond length (d_{av}).

S-2.3 Definition of Selected Physical-Chemical Properties

Relative Total Energy: A crucial aspect of our investigation involves the use of several energetic properties; however, their mathematical definitions were not explicitly outlined in the main manuscript. Therefore, we provided these definitions for clarity and completeness. The mathematical definition of relative total energy (ΔE^i_{tot}) is given by the following equation:

$$\Delta E^i_{tot} = E^i_{tot} - E^{lowest}_{tot} \, , \qquad \text{(Equation S1)}$$

where E^i_{tot} is the total energy of the configuration i under consideration and E^{lowest}_{tot} is the total energy of the configuration with the lowest energy. Therefore, ΔE_{tot} provides insight into the relative stability and, consequently, is a better metric compared to total energy values, that is, smaller values instead of very large numbers.

Binding Energy: The binding energy (E_b) quantifies the interaction strength between the components of a system, for this study, the atoms within the oxide clusters. The

binding energy per atom is calculated as follows:

$$E_b^{cluster} = \frac{(E_{tot}^{cluster} - [aE_{tot}^{\mathrm{Zr}-free\ atoms} + bE_{tot}^{\mathrm{La}-free\ atoms} + cE_{tot}^{\mathrm{Rh}-free\ atoms} + dE_{tot}^{\mathrm{O}-free\ atoms}]}{n},$$

(Equation S2)

where $E_{tot}^{cluster}$ (cluster = $Zr_{16}O_{32}$, $LaZr_{15}O_{32}$, $La_2Zr_{14}O_{31}$, $Rh/Zr_{16}O_{32}$ and $Rh/La_2Zr_{14}O_{31}$) is the total energy of the cluster in the gas-phase, while $a, b, c, dE_{tot}^{free\ atoms}$ (a = 14, 15, 16), (b = 0, 1, 2), (c = 0, 1), (d = 31, 32), and (n = total number of atoms in the system) is the total energy of the free atoms obtained from spin-polarized calculations.

Adsorption and Interaction Energies: Adsorption and interaction energies are essential parameters for evaluating the strength of interaction between an adsorbent, such as oxide clusters, and an adsorbate (e.g. Rh). The adsorption energy (E_{ad}) is determined by the following equation:

$$E_{ad} = E_{tot}^{(\mathrm{Rh}/cluster)} - E_{tot}^{\mathrm{Rh}} - E_{tot}^{cluster}, \quad \text{(Equation S3)}$$

where $E_{tot}^{(\mathrm{Rh}/cluster)}$ is the total energy of the Rh/cluster adsorbed system, E_{tot}^{Rh} is the total energy of the free Rh atom, and $E_{tot}^{cluster}$ is the total energy of the cluster in the gas phase.

The interaction energy (E_int) is determined using the equation:

$$E_{int} = E_{tot}^{\mathrm{Rh}/cluster} - (E_{tot}^{cluster\ frozen} + E_{tot}^{\mathrm{Rh}\ frozen}), \quad \text{(Equation S4)}$$

where, $E_{tot}^{cluster\ frozen}$ (cluster = $Rh/Zr_{16}O_{32}$ and $Rh/La_2Zr_{14}O_{31}$) and $E_{tot}^{\mathrm{Rh}\ frozen}$ are, in the frozen geometry of the adsorbed system, the total energies of the cluster and the free Rh atom, respectively. Thus, E_{ad} and E_{int} are mathematical related as follows:

$$E_{ad} = E_{int} + \Delta E_{tot}^{\mathrm{cluster}} + \Delta E_{tot}^{\mathrm{Rh}}, \quad \text{(Equation S5)}$$

where, $\Delta E_{tot}^{\mathrm{cluster}}$ and $\Delta E_{tot}^{\mathrm{Rh}}$:

$$\Delta E_{tot}^{\mathrm{Rh}} = E_{tot}^{\mathrm{Rh}\ frozen} - E_{tot}^{\mathrm{Rh}\ lowest}, \quad \text{(Equation S6)}$$

$$\Delta E_{tot}^{\mathrm{cluster}} = E_{tot}^{\mathrm{cluster}\ frozen} - E_{tot}^{\mathrm{cluster}\ lowest}. \quad \text{(Equation S7)}$$

Structural Parameters: Following the approach used in several previous studies,[4,5] we evaluated the most relevant structural properties using the effective coordination concept.[6,7]

As a result, we obtained the weighted bond length (d_{av}) and the effective coordination number (ECN^i) for each atom i within the cluster. In this method, the weighted values are calculated using an exponential function to determine the d_{av}^i values, as described below:

$$d_{av}^{i,new} = \frac{\sum_j d_{ij} exp\left[1 - \left(\frac{d_{ij}}{d_{av}^{i,old}}\right)^6\right]}{\sum_j exp\left[1 - \left(\frac{d_{ij}}{d_{av}^{i,old}}\right)^6\right]} , \qquad \text{(Equation S8)}$$

where d_{av}^i is determined using a self-consistent method.

In our implementation, the initial value of $d_{av}^{i,old}$ is set to the shortest distance between the i and j atoms. Convergence is achieved once the condition $\mid d_{av}^{i,new} - d_{av}^{i,old} \mid < 10^{-4}$ Å is satisfied for every atom i in the molecular system. Convergence typically occurs in fewer than 10 steps for complex systems with reduced symmetry. Furthermore, once the d_{av}^i values are determined, the ECN^i results are obtained with the following equation:

$$\text{ECN}^i = \sum_j exp\left[1 - \left(\frac{d_{ij}}{d_{av}^i}\right)^6\right] . \qquad \text{(Equation S9)}$$

Thus, the average values of ECN^i and d_{av}^i, formerly denoted as ECN_{av} and d_{av}, respectively, are obtained as follows:

$$\text{ECN}_{av} = \frac{1}{N} \sum_i \text{ECN}_i , \qquad \text{(Equation S10)}$$

and

$$d_{av} = \frac{1}{N} \sum_i d_{av}^i , \qquad \text{(Equation S11)}$$

where N is the system's total number of atoms. This framework accounts for subtle variations in bond lengths, making it particularly efficient for oxide clusters with reduced symmetry.

Chemical Order Parameter: Another attribute used in this study is the chemical order parameter (σ), which serves to characterize the distribution of Zr, La, Rh, and O atoms within zirconia clusters. This parameter gives insights into the atomic distribution as well as the patterns of interatomic interactions within the cluster. Specifically, it elucidates the interactions among metals, comprising homoatomic metal-metal, heteroatomic metal-oxygen, and homoatomic oxygen-oxygen interactions. Among these interactions, heteroatomic

metal-oxygen interactions are anticipated to be predominant, potentially offering enhanced selectivity and stability.

These interactions can be quantified using the formula below, where M denotes the Zr, La, and Rh atoms:

$$\sigma = \frac{N^{\mathrm{M-M}} + N^{\mathrm{O-O}} - N^{\mathrm{M-O}}}{N^{\mathrm{M-M}} + N^{\mathrm{O-O}} + N^{\mathrm{M-O}}} . \qquad \text{(Equation S12)}$$

where $N^{\mathrm{M-M}}$ indicates the number of homoatomic interactions between metal atoms. $N^{\mathrm{O-O}}$ indicates the number of heteroatomic interactions between oxygen atoms, while $N^{\mathrm{M-O}}$ delineates the total number of heteroatomic interactions between metal M and oxygen O atoms in the cluster, taking into account only the interactions of the nearest neighbor.

S-2.4 Representative Cluster Selection

Starting with a set of distinct chemical clusters, the *k-means*[8] clustering algorithm is used to select representative samples. The *k-means* algorithm effectuates a partitioning of the data set into k clusters through an iterative process of adjusting the centroids and cluster assignments, with the objective of minimizing the sum of squared distances within each cluster.

The *k-means* psudocode is depicted below:

Algorithm 1 K-means Clustering Algorithm

Input: Dataset $\mathcal{X} = \{x_1, x_2, \ldots, x_N\}$, number of clusters k

Output: Cluster assignments $\mathcal{C} = \{C_1, C_2, \ldots, C_k\}$, cluster centroids $\mu_1, \mu_2, \ldots, \mu_k$

Initialize k centroids $\mu_1, \mu_2, \ldots, \mu_k$ randomly from $\mathcal{X}$

repeat

  Assign each sample $x_i \in \mathcal{X}$ to the nearest centroid:

$$C_j = \{x_i : \|x_i - \mu_j\|^2 \leq \|x_i - \mu_{j'}\|^2, \forall j' \neq j\}$$

  Update each centroid as the mean of its assigned samples:

$$\mu_j = \frac{1}{|C_j|} \sum_{x_i \in C_j} x_i$$

until centroids no longer change or maximum iterations are reached

Here, each molecular cluster is represented using the eigenvalues of its Coulomb matrix,[9]

which is defined as following for a molecular system composed of N atoms.

$$C_{ij} = \begin{cases} 0.5Z_i^{2.4} & \text{if } i = j, \\ \frac{Z_i Z_j}{\|\mathbf{r}_i - \mathbf{r}_j\|} & \text{if } i \neq j, \end{cases}$$

where Z_i is the atomic number of the i-th atom, $\mathbf{r}_i$ is its Cartesian coordinate site, and $\|\mathbf{r}_i - \mathbf{r}_j\|$ represents the Euclidean distance between atoms i and j. The eigenvalues of the Coulomb matrix provide a compact representation of each chemical cluster for clustering purposes. Once the clustering process is complete, a sample is selected for each cluster. In our case, we select the molecular cluster closest to the centroid of the group. The Python code used in our experiments is available at `https://github.com/quiles/Adsorption_Clus`.

S-3 Additional Data for the Oxide Clusters

In the following, we provide additional data, including representative optimized molecular configurations and structural parameters for the following clusters: $Zr_{16}O_{32}$, $LaZr_{15}O_{32}$, $La_2Zr_{14}O_{31}$, Rh/$Zr_{16}O_{32}$ and Rh/$La_2Zr_{14}O_{31}$.

S-3.1 $Zr_{16}O_{32}$

To construct the $Zr_{16}O_{32}$ clusters, we selected five $(ZrO_2)_{15}$ clusters from the work of Zibordi-Besse et al.,[10] including the putative global minimum configuration (pGMC) for the $(ZrO_2)_{15}$ cluster. For each selected $(ZrO_2)_{15}$ cluster, thousands of configurations were generated by adsorbing a ZrO_2 fragment onto the clusters. The ZrO_2 fragment was initially positioned approximately 1.5 Å above the clusters in random orientations.

To efficiently manage the large dataset, the *k-means* clustering algorithm was used, reducing the number of configurations from thousands to approximately 20 configurations for each cluster, producing a total of around 100 initial test configurations for $Zr_{16}O_{32}$. Furthermore, symmetrical structures derived from solid crystalline fragments were included, together with the structure proposed by Puigdollers.[11] The Puigdollers structure was selected due to its preserved symmetry (Sym) derived from the tetragonal structure of ZrO_2 (t-ZrO_2).

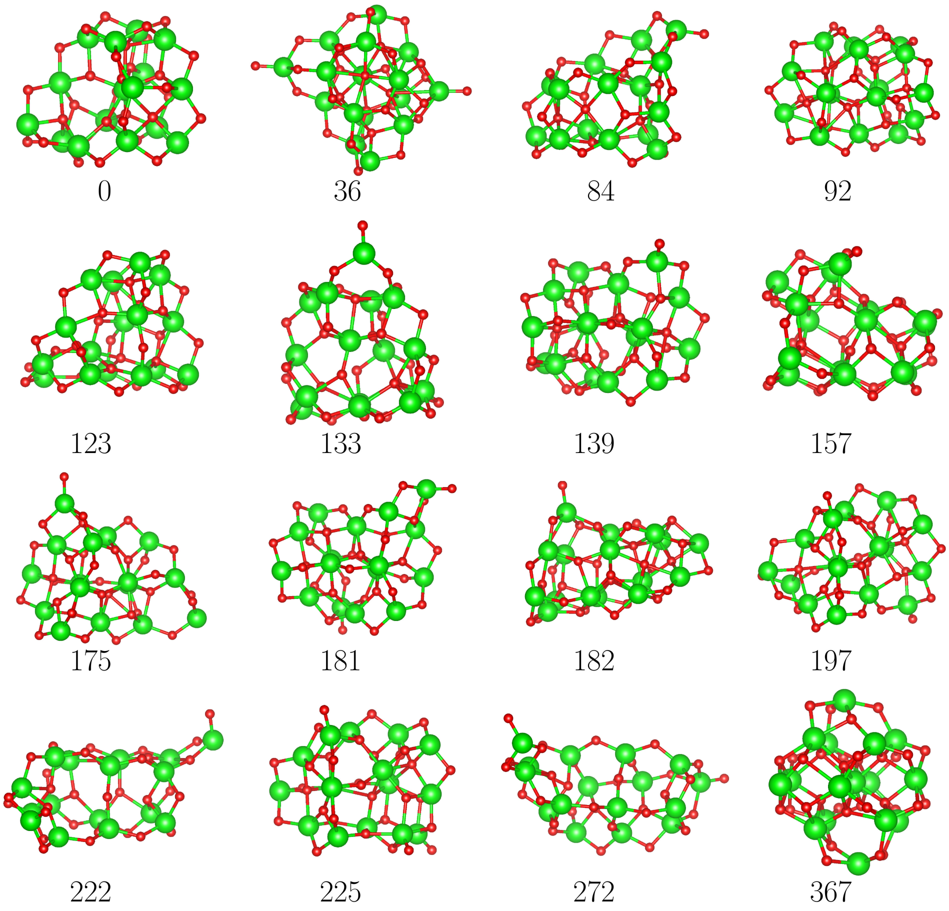

Figure S-3: Representative set of optimized $Zr_{16}O_{32}$ configurations. The numbers below each structure is the relative total energy ($\Delta E_{tot} = E^{i}_{tot} - E^{lowest}_{tot}$) given in meV per formula unit, where E^{lowest}_{tot} is the total energy of the lowest energy configuration (putative global minimum configuration - pGMC), and the last structure is derived Puigdollers (Sym).[11]

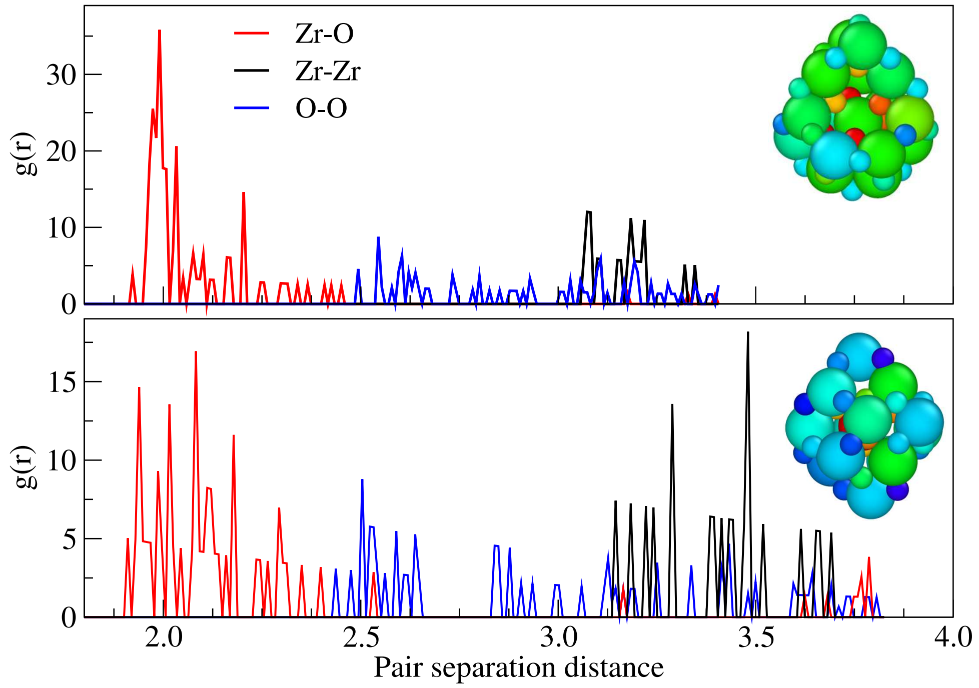

Figure S-4: Partial radial function g(r) of the $Zr_{16}O_{32}$ to Zr−O (red), Zr−Zr (black) and O−O (blue). The curls represent the coordination analyzed through the partial radial function, where the colors next to blue are less coordinated than the colors next to red.

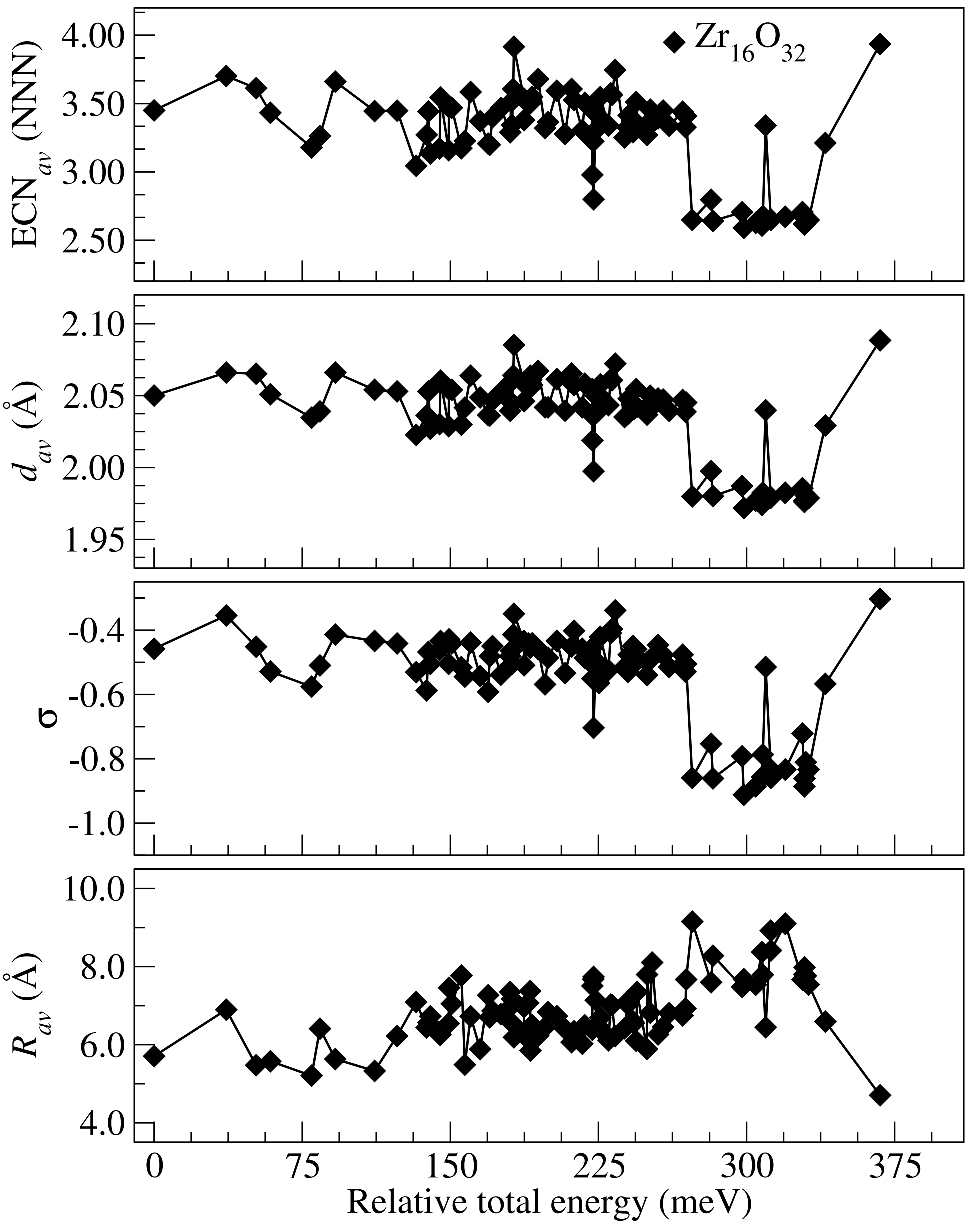

Figure S-5: Characterization of the structural parameters for the $Zr_{16}O_{32}$ configurations: Average effective coordination number (ECN_{av}) in number of nearest neighbors (NNN), average weighted bond length (d_{av}), chemical order parameter (σ), and average cluster radius (R_{av}).

S-3.2 $LaZr_{15}O_{32}$

To generate the $LaZr_{15}O_{32}$ cluster, we started the design process using the pGMC and Sym structures of the $Zr_{16}O_{32}$ cluster. First, a Zr atom was replaced with a La atom in all possible configurations within the $Zr_{16}O_{32}$ clusters, resulting in a total of 16 new structures.

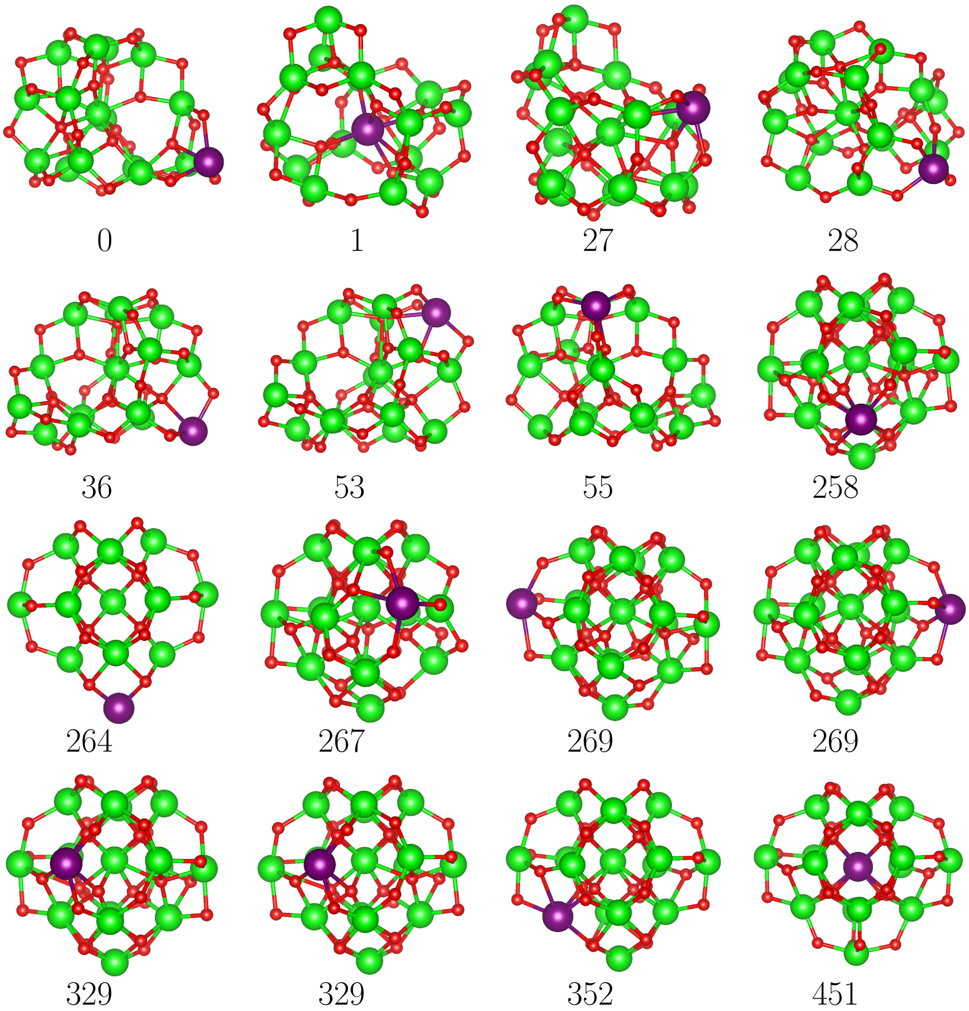

Figure S-6: Representative set of optimized $LaZr_{15}O_{32}$ configurations. The numbers below each structure is the relative total energy ($\Delta E_{tot} = E^{i}_{tot} - E^{lowest}_{tot}$) given in meV per formula unit, where E^{lowest}_{tot} is the total energy of the lowest energy configuration (putative global minimum configuration).

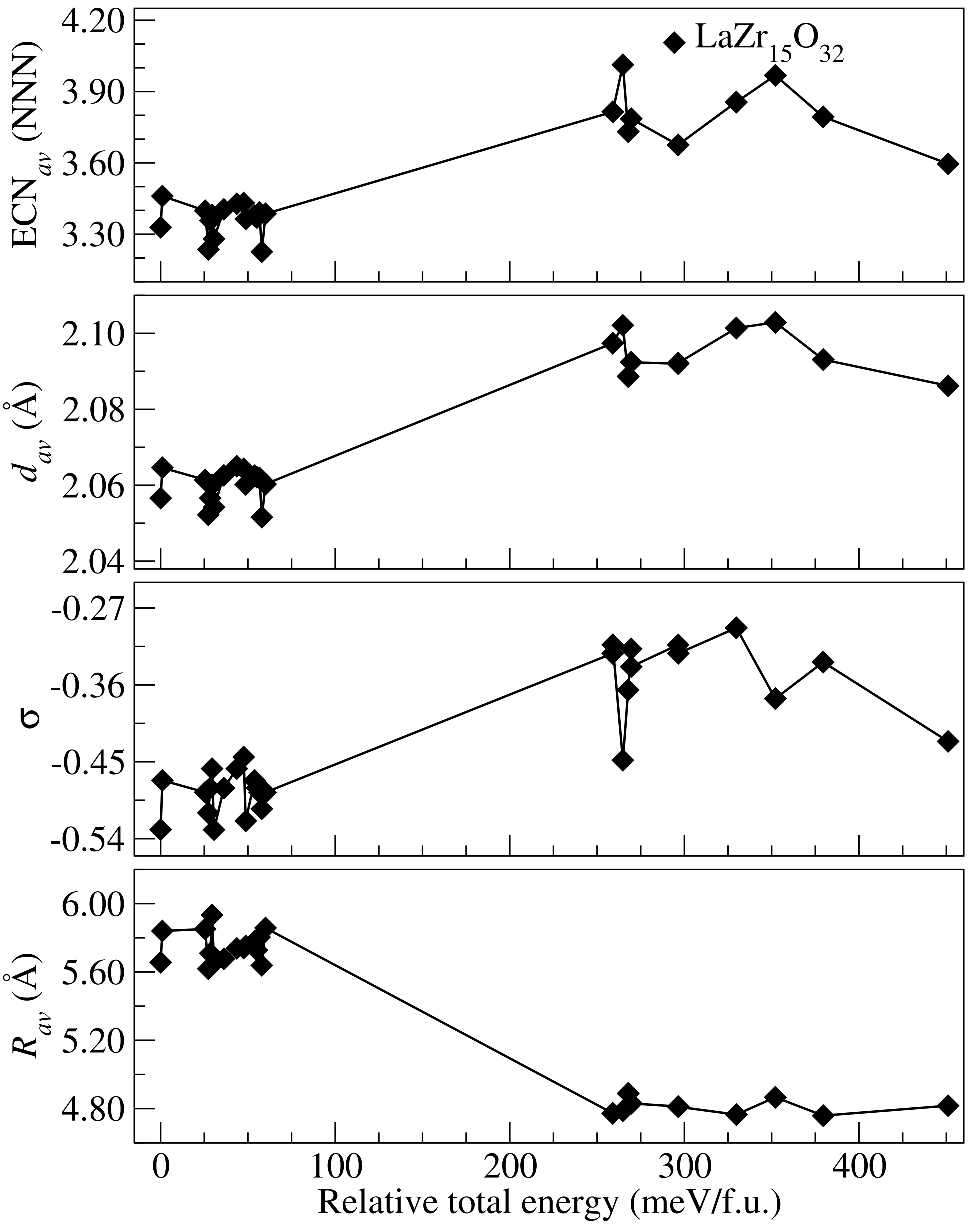

Figure S-7: Characterization of the structural parameters for the $LaZr_{15}O_{32}$ configurations: Average effective coordination number (ECN_{av}) in number of nearest neighbors (NNN), average weighted bond length (d_{av}), chemical order parameter (σ), and average cluster radius (R_{av}).

S-3.3 $La_2Zr_{14}O_{31}$

For generating the $La_2Zr_{14}O_{31}$ clusters, we started with the pGMC-derived structure $Zr_{16}O_{32}$ and the Sym dataset. Initially, two Zr atoms are replaced with La atoms in all possible configurations within $Zr_{16}O_{32}$, using a custom script to automate the substitutions. This step generates120 structures for each initial cluster configuration. To refine this large dataset, the *k-means* clustering algorithm is applied, reducing the set to 32 representative structures. For

each selected structure, one O atom closest to the La atoms is removed to preserve stoichiometry, resulting in the $La_2Zr_{14}O_{31}$ cluster. This stoichiometry is in alignment with that of lanthanum oxide (La_2O_3), as described by the chemical equation: $La_2O_3 + (ZrO_2)_{14} \longrightarrow La_2Zr_{14}O_{31}$.

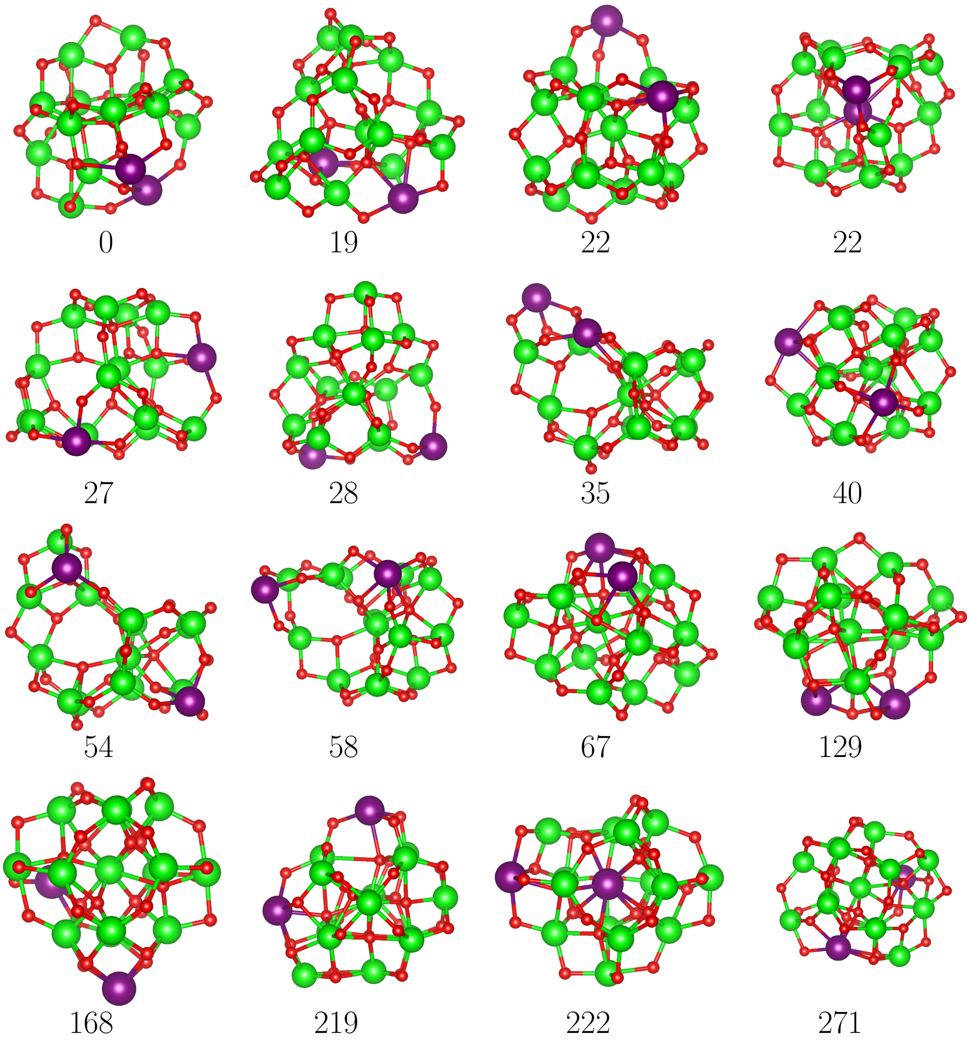

Figure S-8: Representative set of optimized $La_2Zr_{14}O_{31}$ configurations. The numbers below each structure is the relative total energy ($\Delta E_{tot} = E^{i}_{tot} - E^{lowest}_{tot}$) given in meV per formula unit, where E^{lowest}_{tot} is the total energy of the lowest energy configuration (putative global minimum configuration).

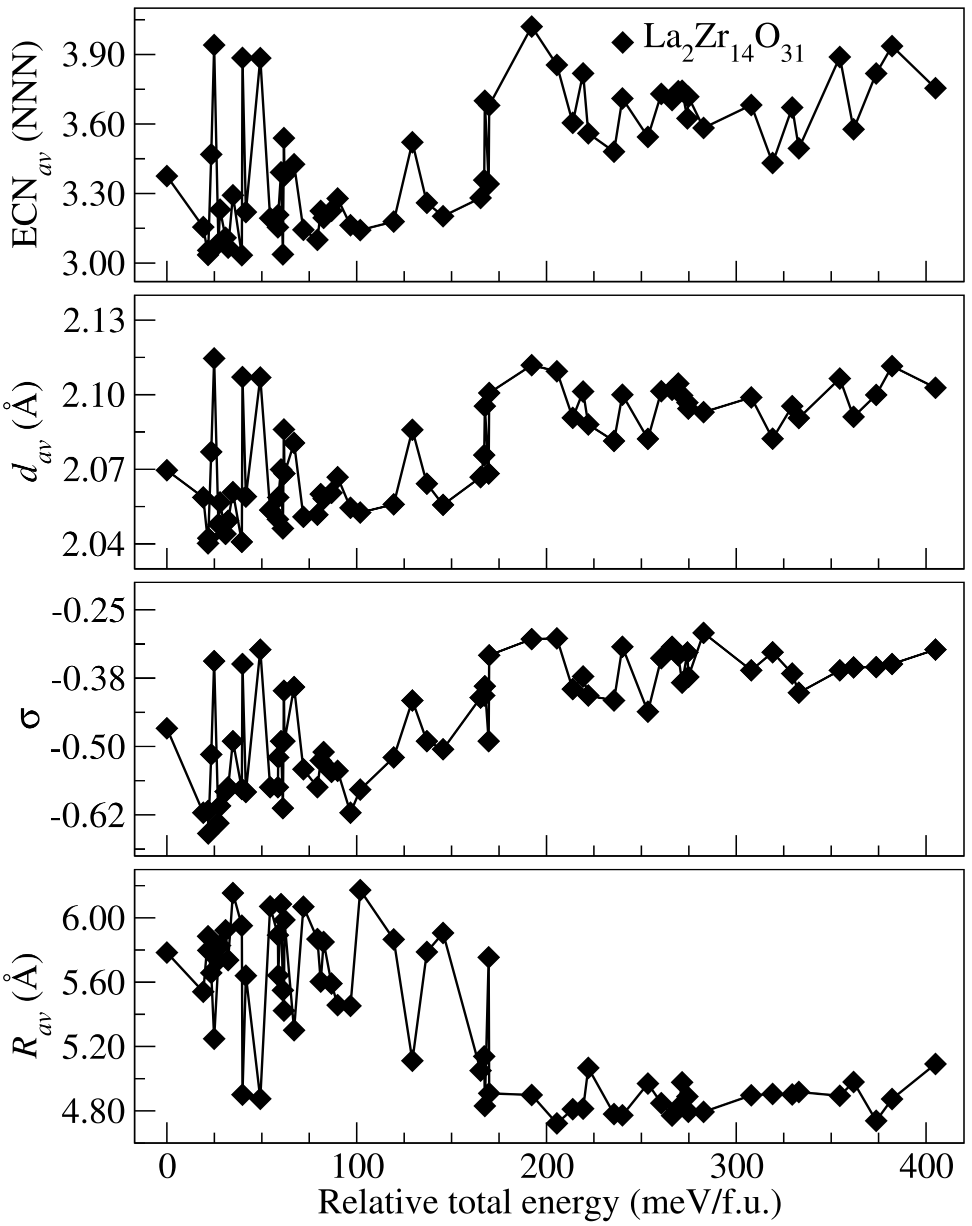

Figure S-9: Characterization of the structural parameters for the $La_2Zr_{14}O_{31}$ configurations: Average effective coordination number (ECN_{av}) in number of nearest neighbors (NNN), average weighted bond length (d_{av}), chemical order parameter (σ), and average cluster radius (R_{av}).

S-3.4 Rh/$Zr_{16}O_{32}$

Initially, the cluster adsorption algorithm, developed in our group and used in previous studies,[4,5,12] was used to generate the adsorbed Rh/$Zr_{16}O_{32}$ systems. In this approach, the $Zr_{16}O_{32}$ structure is kept frozen, and the adsorbate Rh free atom is randomly placed at a specific distance above its surface, generating millions of test configurations for Rh/$Zr_{16}O_{32}$. Subsequently, to refine this dataset, the *k-means* clustering algorithm was applied to reduce it to

32 representative structures of $Rh/Zr_{16}O_{32}$.

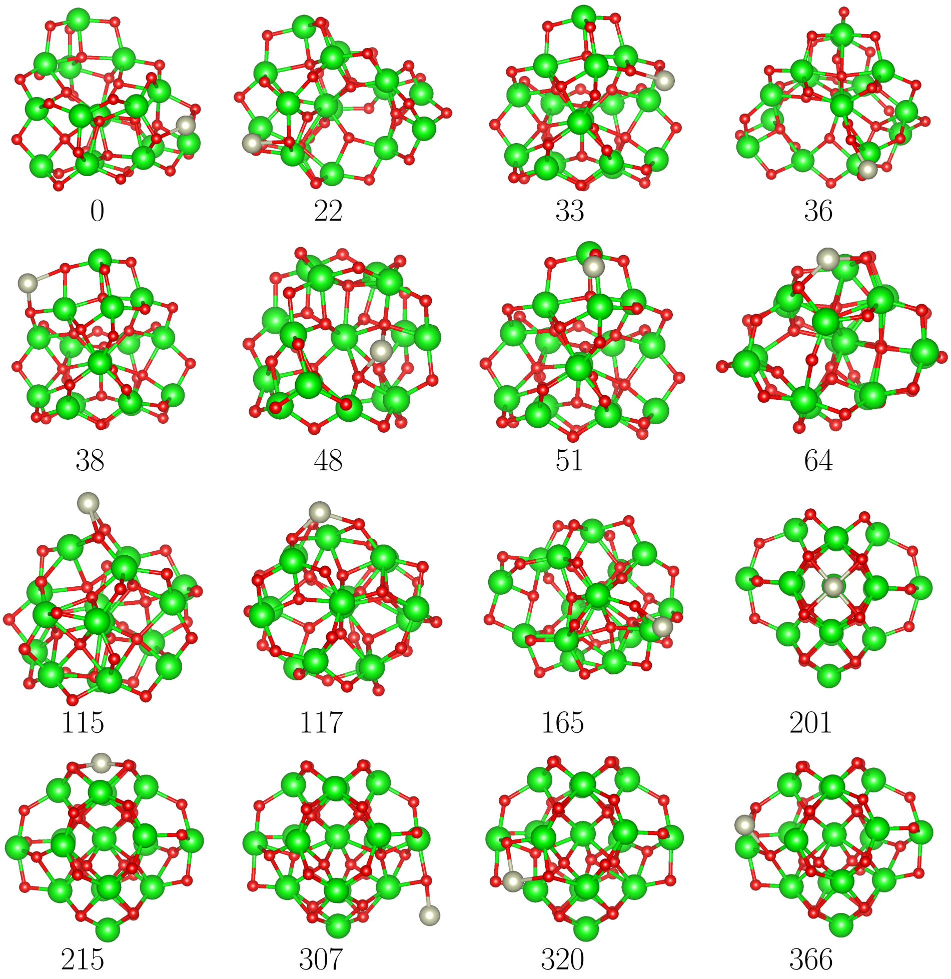

Figure S-10: Representative set of optimized $Rh/Zr_{16}O_{32}$ configurations. The numbers below each structure is the relative total energy ($\Delta E_{tot} = E^{i}_{tot} - E^{lowest}_{tot}$) given in meV per formula unit, where E^{lowest}_{tot} is the total energy of the lowest energy configuration (putative global minimum configuration).

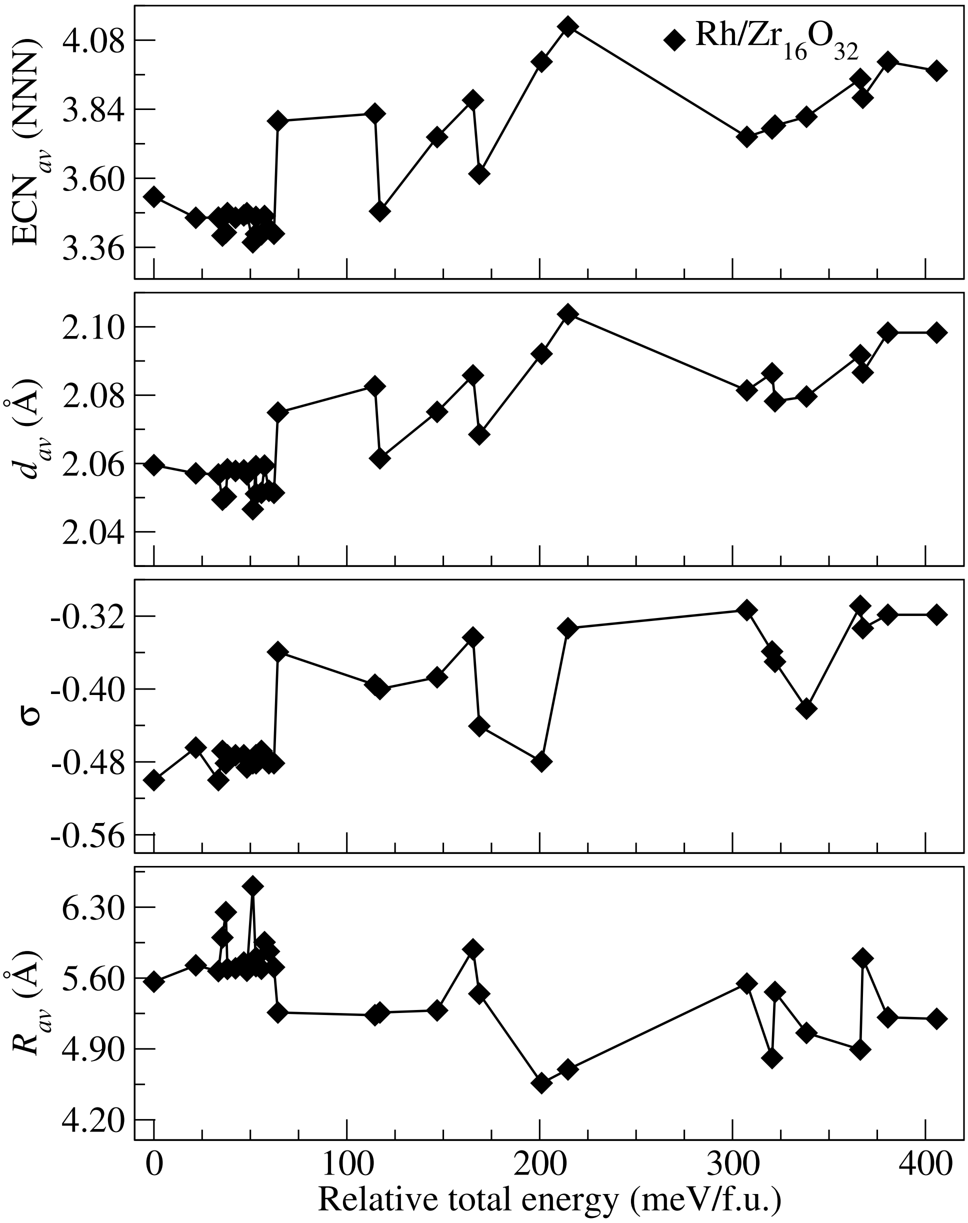

Figure S-11: Characterization of the structural parameters for the $Rh/Zr_{16}O_{32}$ configurations: Average effective coordination number (ECN_{av}) in number of nearest neighbors (NNN), average weighted bond length (d_{av}), chemical order parameter (σ), and average cluster radius (R_{av}).

S-3.5 $Rh/La_2Zr_{14}O_{31}$

As described in the previous section, $Rh/Zr_{16}O_{32}$ the same framework was used to generate $Rh/La_2Zr_{14}O_{31}$. However, the pGMC and Sym structures of $La_2Zr_{14}O_{31}$ were used as a starting point. After applying the framework, i.e., cluster adsorption and *k-means*, 32 representative structures of $Rh/La_2Zr_{14}O_{31}$ were obtained.

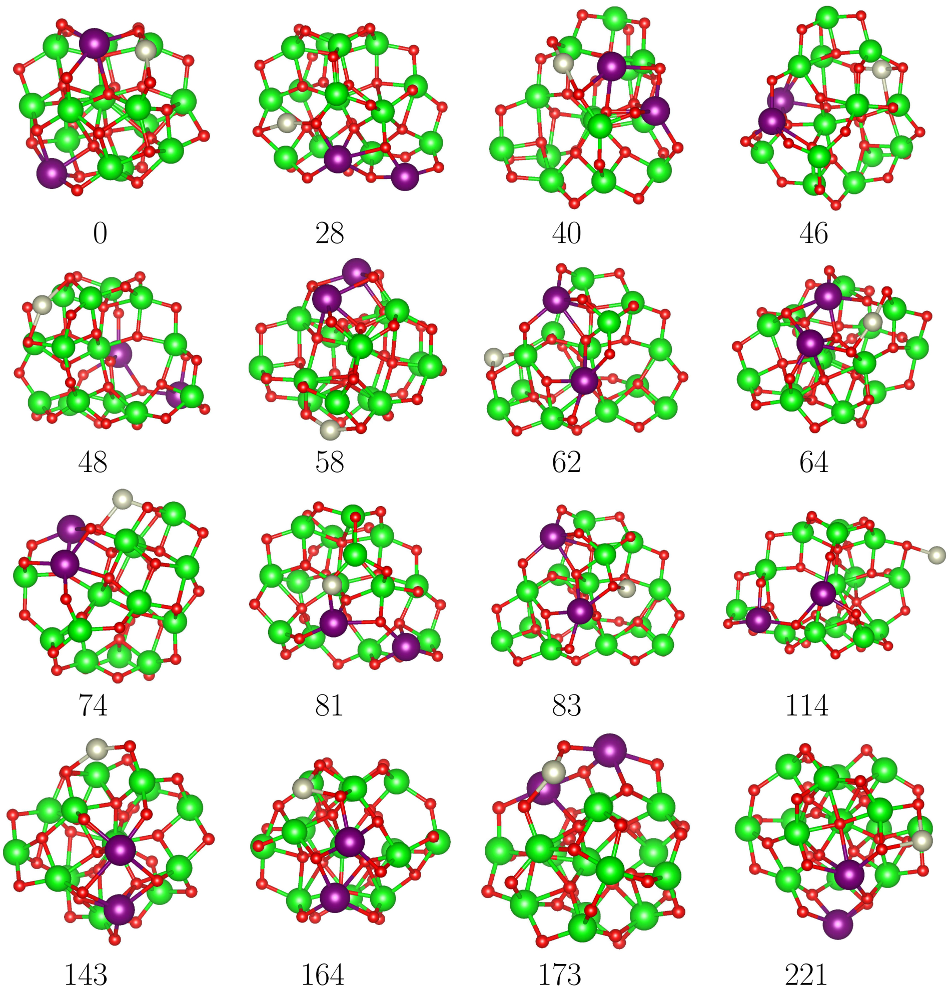

Figure S-12: Representative set of optimized $Rh/La_2Zr_{14}O_{31}$ configurations. The numbers below each structure is the relative total energy ($\Delta E_{tot} = E^{i}_{tot} - E^{lowest}_{tot}$) given in meV per formula unit, where E^{lowest}_{tot} is the total energy of the lowest energy configuration (putative global minimum configuration).

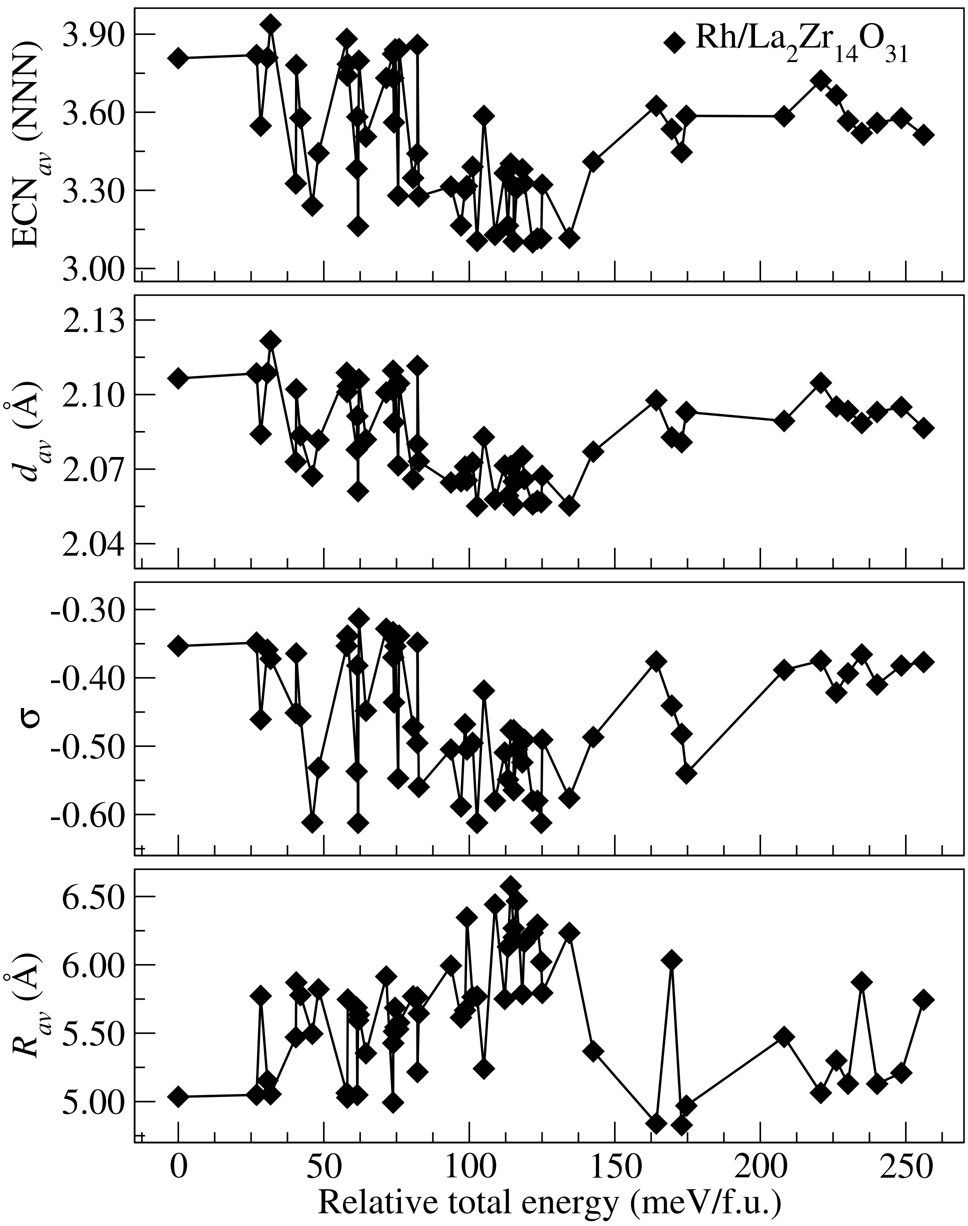

Figure S-13: Characterization of the structural parameters for the Rh/$La_2Zr_{14}O_{31}$ configurations: Average effective coordination number (ECN$_{av}$) in number of nearest neighbors (NNN), average weighted bond length (d_{av}), chemical order parameter (σ), and average cluster radius R_{av}.

S-4 Atomic Positions of the Lowest Energy Configurations

$Zr_{16}O_{32}$ - pGMC, $E_{tot} = -1\,646\,157.228\,133\,73\,\mathrm{eV}$, $\Delta E_{tot} = 0\,\mathrm{meV}$

Zr	1.6649210000000001	1.9489420000000000	−2.6257869999999999
O	0.1375210000000000	1.6910019999999999	−3.8658809999999999
O	0.6438290000000000	1.6283069999999999	−0.9062620000000000
Zr	−0.1124760000000000	2.2878910000000001	4.8068049999999998
O	−1.9507509999999999	1.8148599999999999	4.2752509999999999
O	1.5149830000000000	1.1651849999999999	4.8549400000000000
Zr	0.6981470000000000	3.8808379999999998	2.2640030000000002
O	0.7898610000000000	4.9908780000000004	0.6137670000000000
O	0.2336000000000000	4.1372869999999997	4.2251640000000004
Zr	−1.6513460000000000	2.0629510000000000	−3.0567489999999999
O	−1.5285280000000001	3.7219150000000001	−1.9796769999999999
O	−3.3468170000000002	1.1947779999999999	−3.5819200000000002
Zr	−3.4581350000000000	0.6253910000000000	0.8331730000000001
O	−3.3317950000000001	2.4000759999999999	1.7164900000000001
O	−4.5760660000000000	0.3257800000000000	−0.7898430000000000
Zr	−1.9809319999999999	1.7686070000000000	2.2465320000000002
O	−1.0597380000000001	3.2291129999999999	1.1098779999999999
O	−3.7748870000000001	1.0547560000000000	1.8764920000000000
Zr	−3.3564080000000001	0.3742860000000000	−2.3267810000000000
Zr	0.0292880000000000	3.7412269999999999	−0.7325370000000000
O	−2.1805200000000000	1.6317070000000000	−0.6483690000000000
O	−1.6668570000000000	0.6419560000000000	−1.6658759999999999
O	0.1390180000000000	1.8719800000000000	2.8293070000000000
O	−2.9992009999999998	2.0378300000000000	−3.3476889999999999
Zr	−1.5861190000000001	3.0222359999999999	−2.3954819999999999
O	1.5233449999999999	3.8002899999999999	−2.1154280000000001
O	−0.0045290000000000	3.8348680000000002	−3.3234059999999999
Zr	−1.8609100000000001	3.3966759999999998	0.7901620000000000
O	−0.5609110000000000	4.6317250000000003	1.7249840000000001
O	−2.0946959999999999	4.2865300000000000	−0.9422110000000000
Zr	2.1637610000000000	1.0919360000000000	2.9111490000000000
O	−1.4559240000000000	0.1276150000000000	1.1660560000000000
O	2.4364629999999998	2.9498869999999999	2.2631790000000001
Zr	3.2202660000000001	1.1686589999999999	1.0797280000000000
O	1.4624320000000000	0.0400430000000000	1.2948930000000001
O	3.7685599999999999	0.0305030000000000	2.5816010000000000
Zr	3.8014139999999998	0.8566020000000000	−2.0311889999999999
Zr	−0.0791390000000000	0.4092290000000000	−0.3092390000000000
Zr	1.4828870000000001	2.9801679999999999	−2.3310800000000000
Zr	1.1685749999999999	3.9163839999999999	1.0482180000000001
O	4.7031780000000003	1.0569470000000001	−0.3038030000000000
O	3.3870740000000001	0.9334500000000000	−2.7524950000000001
O	3.2192330000000000	2.3962699999999999	−3.1364169999999998
O	2.1357610000000000	1.1668590000000001	−0.9554140000000000
O	−0.0581980000000000	1.6176649999999999	−2.0561020000000001
O	1.6823999999999999	4.2410620000000003	−0.8090950000000000
O	−0.0235530000000000	2.3543479999999999	0.6494030000000000
O	2.6919209999999998	2.9338069999999998	1.8275560000000000

References

1 Blum, V.; Gehrke, R.; Hanke, F.; Havu, P.; Havu, V.; Ren, X.; Reuter, K.; Scheffler, M. *Ab initio* Molecular Simulations With Numeric Atom-Centered Orbitals. *Comput. Phys. Commun.* **2009**, *180*, 2175–2196, DOI: 10.1016/j.cpc.2009.06.022.

2 Havu, V.; Blum, V.; Havu, P.; Scheffler, M. Efficient Integration for all-Electron Electronic Structure Calculation Using Numeric Basis Functions. *J. Comput. Phys.* **2009**, *228*, 8367–8379, DOI: 10.1016/j.jcp.2009.08.008.

3 Felício-Sousa, P.; Mucelini, J.; Zibordi-Besse, L.; Andriani, K. F.; Seminovski, Y.; Prati, R. C.; Da Silva, J. L. F. *Ab Initio* Insights Into the Structural, Energetic, Electronic, and Stability Properties of Mixed $Ce_nZr_{15-n}O_{30}$ Nanoclusters. *Phys. Chem. Chem. Phys.* **2019**, *21*, 26637–26646, DOI: 10.1039/c9cp04762j.

4 Felício-Sousa, P.; Andriani, K. F.; Da Silva, J. L. F. Ab initio investigation of the role of the d-states occupation on the adsorption properties of H_2, CO, CH_4 and CH_3OH on the Fe_{13}, Co_{13}, Ni_{13} and Cu_{13} clusters. *Phys. Chem. Chem. Phys.* **2021**, *23*, 8739–8751, DOI: 10.1039/D0CP06091G.

5 Andriani, K. F.; Felício-Sousa, P.; Morais, F. O.; Da Silva, J. L. F. Role of Quantum-size Effects on the Dehydrogenation of CH_4 on 3*d* TMn Clusters: DFT Calculations Combined with Data Mining. *Catal. Sci. Technol.* **2021**, 916–926, DOI: 10.1039/D1CY01785C.

6 Hoppe, R. The Coordination Number - An “Inorganic Chameleon”. *Angew. Chem. Int. Ed.* **1970**, *9*, 25–34, DOI: 10.1002/anie.197000251.

7 Da Silva, J. L. F. Effective Coordination Concept Applied for Phase Change $(GeTe)_m(Sb_2Te_3)_n$ Compounds. *J. Appl. Phys.* **2011**, *109*, 023502, DOI: 10.1063/1.3533422.

8 Jain, A. K. Data Clustering: 50 Years Beyond K-Means. *Pattern Recognit. Lett.* **2010**, *31*, 651–666, DOI: 10.1016/j.patrec.2009.09.011.

9 Hansen, K.; Montavon, G.; Biegler, F.; Fazli, S.; Rupp, M.; Scheffler, M.; von Lilienfeld, O. A.; Tkatchenko, A.; Müller, K.-R. Assessment and Validation of Machine Learning Methods for Predicting Molecular Atomization Energies. *J. Chem. Theory Comput.* **2013**, *9*, 3404–3419, DOI: 10.1021/ct400195d.

10 Zibordi-Besse, L.; Seminovski, Y.; Rosalino, I.; Guedes-Sobrinho, D.; Da Silva, J. L. F. Physical and Chemical Properties of Unsupported $(MO_2)_n$ Clusters for M = Ti, Zr, or Ce and $n = 1 - 15$: A Density Functional Theory Study Combined With the Tree-growth Scheme and Euclidean Similarity Distance Algorithm. *J. Phys. Chem. C* **2018**, *122*, 27702–27712, DOI: 10.1021/acs.jpcc.8b08299.

11 Puigdollers, A. R.; Illas, F.; Pacchioni, G. ZrO_2 Nanoparticles: A Density Functional Theory Study of Structure, Properties and Reactivity. *Rend. Lincei Sci. Fis. Nat.* **2016**, *28*, 19–27, DOI: 10.1007/s12210-016-0591-x.

12 Andriani, K. F.; Mucelini, J.; Da Silva, J. L. F. Methane Dehydrogenation on 3*d* 13-Atom Transition-Metal Clusters: A Density Functional Theory Investigation Combined with Spearman Rank Correlation Analysis. *Fuel* **2020**, *275*, 117790, DOI: 10.1016/j.fuel.2020.117790.

$Zr_{16}O_{32}$ - Sym, $E_{tot} = -1\,646\,151.339\,767\,98$ eV, $\Delta E_{tot} = 123$ meV

O	10.7052189999999996	8.8473369999999996	11.1933450000000008
O	10.5275630000000007	12.8476280000000003	11.3171099999999996
O	10.3179599999999994	12.1458790000000008	16.1905339999999995
O	9.7530830000000002	15.5621270000000003	10.3013460000000006
O	14.4326439999999998	9.6240900000000007	11.1421109999999999
O	14.2996499999999997	12.4984540000000006	11.2989219999999992
O	14.8728110000000004	12.7275200000000002	16.2756669999999986
O	14.2028759999999998	16.1137510000000006	11.0975300000000008
O	8.8470580000000005	10.7055380000000007	13.8063369999999992
O	9.6242249999999991	14.4328599999999998	13.8578419999999998
O	12.1458779999999997	10.3182019999999994	8.8093280000000007
O	12.8473620000000004	10.5274439999999991	13.6828090000000007
O	12.7279940000000007	14.8729809999999993	8.7244919999999997
O	12.4985769999999992	14.2995769999999993	13.7011930000000000
O	15.5617180000000008	9.7526229999999998	14.6986810000000006
O	16.1138550000000009	14.2024069999999991	13.9027840000000005
O	10.7758889999999994	8.8927820000000004	13.7795980000000000
O	10.4236369999999994	12.5816750000000006	8.7420740000000006
O	11.0097000000000005	12.2183360000000008	13.8126320000000007
O	10.9545899999999996	16.6313770000000005	12.6389180000000003
O	13.9894370000000006	7.9777250000000004	12.9760120000000008
O	14.3738679999999999	12.5600090000000009	8.7699359999999995
O	14.2479849999999999	12.5118480000000005	13.8166410000000006
O	14.0582100000000008	16.2305290000000007	14.2039819999999999
O	8.8926710000000000	10.7763469999999995	11.2201000000000004
O	7.9779130000000000	13.9899470000000008	12.0238180000000003
O	12.2182689999999994	11.0097980000000000	11.1872779999999992
O	12.5812580000000001	10.4234050000000007	16.2578269999999989
O	12.5121140000000004	14.2480600000000006	11.1834729999999993
O	12.5600020000000008	14.3736219999999992	16.2301859999999998
O	16.6312250000000006	10.9541540000000008	12.3612439999999992
O	16.2308009999999996	14.0579070000000002	10.7963339999999999
Zr	11.1207039999999999	14.2681799999999992	9.5934249999999999
Zr	11.0045009999999994	13.7871059999999996	15.2963339999999999
Zr	13.7871249999999996	11.0045190000000002	9.7036630000000006
Zr	14.2678829999999994	11.1203459999999996	15.4066080000000003
Zr	14.3163060000000009	14.3396360000000005	10.0285569999999993
Zr	14.3396939999999997	14.3159620000000007	14.9716640000000005
Zr	9.2598699999999994	9.2602220000000006	12.4998170000000002
Zr	9.3151750000000000	15.6399749999999997	12.1878679999999999
Zr	15.6396250000000006	9.3148169999999997	12.8121360000000006
Zr	10.6141199999999998	11.0352770000000007	9.9167810000000003
Zr	11.0349500000000003	10.6141109999999994	15.0830409999999997
Zr	9.0900090000000002	12.5415469999999996	12.6648399999999999
Zr	12.5411920000000006	9.0900119999999998	12.3349740000000008
Zr	12.6041670000000003	12.6041550000000004	12.5000090000000004
Zr	12.9597350000000002	16.0620979999999989	12.6093279999999996
Zr	16.0621580000000002	12.9593589999999992	12.3909129999999994

$LaZr_{15}O_{32}$ - D-pGMC, $E_{tot} = -1\,782\,567.618\,325\,27\,\text{eV}$, $\Delta E_{tot} = 0\,\text{meV}$

Zr	1.6116029999999999	−2.2645080000000002	−2.8243550000000002
O	0.0362550000000000	−1.9335050000000000	−3.9725649999999999
O	0.6754070000000000	−1.9728049999999999	−1.0934200000000001
Zr	−0.0931100000000000	−2.3333610000000000	4.7981800000000003
O	−1.9371870000000000	−1.8577889999999999	4.2788449999999996
O	1.5572379999999999	−1.2470900000000000	4.9726460000000001
Zr	0.7595720000000000	−3.8143530000000001	2.2115499999999999
O	0.8275480000000000	−5.0114070000000002	0.6250160000000000
O	0.2475480000000000	−4.1552559999999996	4.1368919999999996
Zr	−1.7662709999999999	−2.2017050000000000	−3.1189719999999999
O	−1.6493480000000000	−3.8256429999999999	−1.9981130000000000
O	−3.4594450000000001	−1.2920990000000001	−3.6483569999999999
Zr	−3.4499420000000001	0.6437920000000000	0.8399870000000000
O	−3.3175919999999999	2.4526409999999998	1.6767399999999999
O	−4.5527430000000004	0.2706870000000000	−0.8046110000000000
Zr	−1.9727049999999999	−1.7462660000000001	2.2561740000000001
O	−0.9868310000000000	−3.1559430000000002	1.0630599999999999
O	−3.7854719999999999	−1.0674300000000001	1.8478960000000000
Zr	−3.4268480000000001	0.2937900000000000	−2.4022340000000000
Zr	−0.0063820000000000	−3.9268440000000000	−0.8283020000000000
O	−2.2017760000000002	1.6729330000000000	−0.7436700000000001
O	−1.7505459999999999	−0.7249280000000000	−1.8065730000000000
O	0.2102250000000000	−1.8092310000000000	2.8592819999999999
O	−3.0918019999999999	2.0347599999999999	−3.3482289999999999
Zr	−1.7331120000000000	3.0035520000000000	−2.3187050000000000
O	1.4045500000000000	−4.1255100000000002	−2.3027910000000000
O	−0.0076670000000000	3.8721809999999999	−3.2154099999999999
Zr	−1.8609230000000001	3.5209280000000001	0.8005040000000000
O	−0.5770920000000000	4.7471170000000003	1.7692450000000000
O	−2.2060379999999999	4.3685349999999996	−0.9399920000000000
Zr	2.2923589999999998	−1.0972759999999999	3.0688780000000002
O	−1.5181979999999999	−0.0150700000000000	1.3464540000000000
O	2.5356139999999998	−2.9259059999999999	2.3509220000000002
Zr	3.3187090000000001	1.2162370000000000	1.2765059999999999
O	1.7014250000000000	0.0226660000000000	1.4185000000000001
O	3.8555839999999999	0.1524500000000000	2.8400330000000000
Zr	3.7395830000000001	0.8253210000000000	−1.9545889999999999
La	0.0091270000000000	0.3122990000000000	−0.3738750000000000
Zr	1.6337600000000001	3.1221179999999999	−2.1467909999999999
Zr	1.1830660000000000	4.0708469999999997	1.0766320000000000
O	4.6688070000000002	0.8860780000000000	−0.2315180000000000
O	3.1426289999999999	−0.9189530000000000	−2.6893729999999998
O	3.3101170000000000	2.4797030000000002	−3.0056620000000001
O	2.1707429999999999	1.4573769999999999	−0.8861070000000000
O	0.0072070000000000	1.8295669999999999	−2.4261040000000000
O	1.8146650000000000	4.5138189999999998	−0.7459540000000000
O	−0.0145900000000000	2.6389649999999998	0.4358360000000000
O	2.6522770000000002	3.0145110000000002	1.8764940000000001

$LaZr_{15}O_{32}$ - D-Sym, $E_{tot} = -1\,782\,569.124\,186\,15\,\mathrm{eV}$, $\Delta E_{tot} = 86\,\mathrm{meV}$

Zr	11.0801960000000008	14.1525180000000006	9.6640949999999997
Zr	11.0207379999999997	13.8293429999999997	15.3509309999999992
Zr	13.8616370000000000	11.0523710000000008	9.6910270000000001
Zr	14.3257049999999992	11.1660459999999997	15.4333600000000004
Zr	14.3379530000000006	14.2800790000000006	9.9872669999999992
Zr	14.2810930000000003	14.4034920000000000	15.0209910000000004
Zr	9.2787509999999997	9.4070999999999998	12.3681809999999999
Zr	9.4559630000000006	15.3272949999999994	12.0798109999999994
Zr	15.2915799999999997	9.4611699999999992	12.9324580000000005
Zr	10.7288820000000005	10.6396420000000003	9.6206180000000003
La	10.6623940000000008	10.4118689999999994	15.5749770000000005
Zr	9.0208230000000000	12.5573329999999999	12.9583639999999995
Zr	12.5102320000000002	9.0251719999999995	12.0813419999999994
Zr	12.5486409999999999	12.5826279999999997	12.5752290000000002
Zr	12.9461440000000003	16.0219029999999982	12.4900289999999998
Zr	16.0078220000000009	12.9427260000000004	12.4779040000000006
O	10.6044870000000007	8.8209350000000004	10.8349530000000005
O	10.4264849999999996	13.5082369999999994	11.7504439999999999
O	10.2521609999999992	12.4155359999999995	16.4458440000000010
O	9.7232430000000001	15.5168739999999996	10.1211889999999993
O	14.3806449999999995	9.5348600000000001	10.8860320000000002
O	14.2727590000000006	12.4586629999999996	11.3135060000000003
O	14.8833420000000007	12.8358559999999997	16.3129179999999998
O	14.1642560000000000	16.1524789999999996	10.9238470000000003
O	8.5716970000000003	10.6062360000000009	13.8022259999999992
O	9.5311810000000001	14.4008760000000002	14.1186570000000007
O	12.3450199999999999	10.2709480000000006	8.5942670000000003
O	13.4284839999999992	10.4116669999999996	13.2859149999999993
O	12.7100000000000009	14.6283820000000002	8.6974370000000008
O	12.4775050000000007	14.3156029999999994	13.7134370000000008
O	15.5696619999999992	9.7093559999999997	14.8610150000000001
O	16.1582089999999994	14.1894720000000003	14.0080279999999995
O	10.8760860000000008	9.0670649999999995	13.4500200000000003
O	10.3358690000000006	12.4358540000000009	8.9523689999999991
O	11.0543429999999994	12.0848320000000005	13.9620970000000000
O	10.9922920000000008	16.5307689999999994	12.5457199999999993
O	13.8498889999999992	7.8573519999999997	12.7550249999999998
O	14.6852959999999992	12.5605200000000004	8.7857699999999994
O	14.2076019999999996	12.6137379999999997	13.8681669999999997
O	14.1332330000000006	16.2376539999999991	14.0498840000000005
O	9.2697459999999996	11.0583419999999997	11.3411779999999993
O	7.8619120000000002	13.9108529999999995	12.2479689999999994
O	12.0903930000000006	11.1371660000000006	11.0173609999999993
O	12.7281200000000005	10.4520280000000003	16.2464489999999984
O	12.6094349999999995	14.2095660000000006	11.1904900000000005
O	12.6228569999999998	14.5913249999999994	16.2483819999999994
O	16.4936720000000001	10.9743359999999992	12.4383879999999998
O	16.2048160000000010	14.1151909999999994	10.9244699999999995

$La_2Zr_{14}O_{31}$ - D-pGMC, $E_{tot} = -1\,916\,936.245\,140\,55\,\text{eV}$, $\Delta E_{tot} = 0\,\text{meV}$

La	1.7370420000000000	−2.4465569999999999	−2.3551210000000000
Zr	0.1586230000000000	−2.5850430000000002	4.7719500000000004
La	0.9426460000000000	−4.1153360000000001	1.8857100000000000
Zr	−1.9465250000000001	−2.5224730000000002	−3.4319430000000000
Zr	−3.2647270000000002	0.5696620000000000	0.7527570000000000
Zr	−1.9628170000000000	−1.8046240000000000	2.5630069999999998
Zr	−3.4631479999999999	0.0345970000000000	−2.6032670000000002
Zr	−1.0529029999999999	−2.5513509999999999	−0.4747090000000000
Zr	−1.5030140000000001	2.8181900000000000	−2.3990649999999998
Zr	−1.7972260000000000	3.4718190000000000	0.7532450000000001
Zr	2.2788499999999998	−1.1065480000000001	2.9246319999999999
Zr	3.2770739999999998	1.4067270000000001	1.3151130000000000
Zr	3.7954620000000001	0.8780600000000000	−1.7959540000000001
Zr	0.1631980000000000	0.6293020000000000	−0.1558560000000000
Zr	1.5721050000000001	3.1001989999999999	−2.2481860000000000
Zr	1.2094410000000000	4.1821529999999996	1.1056999999999999
O	−0.0335600000000000	−2.4771399999999999	−3.8076089999999998
O	0.5173620000000000	−1.1420170000000001	−1.0343059999999999
O	−1.7732019999999999	−2.1244079999999999	4.5246269999999997
O	1.7517540000000000	−1.4088039999999999	4.8577300000000001
O	0.6098890000000000	−3.6562960000000002	−0.4581290000000000
O	0.5395290000000000	−4.3446780000000000	4.1353739999999997
O	−2.0155210000000001	−3.6668430000000001	−1.8649899999999999
O	−3.5740310000000002	−1.4153870000000000	−3.9613010000000002
O	−3.2372610000000002	2.3710179999999998	1.6076870000000001
O	−4.3957670000000002	0.1615500000000000	−0.8471300000000000
O	−1.6514629999999999	−3.3436979999999998	1.3121170000000000
O	−3.5960790000000000	−0.7469900000000000	2.2256369999999999
O	−1.8471810000000000	1.5629260000000000	−0.5305970000000000
O	−1.8941530000000000	−1.0685089999999999	−2.0064620000000000
O	0.2039040000000000	−1.9615000000000000	2.8007379999999999
O	−2.8670520000000002	1.7406860000000000	−3.3803869999999998
O	0.0129750000000000	3.8028140000000001	−3.2796750000000001
O	−0.5982200000000000	4.8711609999999999	1.6243620000000001
O	−2.1283930000000000	4.1558500000000000	−1.0533189999999999
O	−1.3882710000000000	−0.6109580000000000	0.8343130000000000
O	2.7003840000000001	−2.8288840000000000	2.0996910000000000
O	1.5225839999999999	0.2241850000000000	1.4671580000000000
O	3.7936790000000000	0.1967950000000000	2.7700380000000000
O	4.7332239999999999	1.1381209999999999	−0.0806450000000000
O	3.3845239999999999	−0.9366540000000000	−2.3919820000000001
O	3.2747310000000001	2.3860329999999998	−3.0110049999999999
O	2.2125770000000000	1.4615400000000001	−0.6928850000000000
O	0.1472020000000000	1.6228060000000000	−2.0284740000000001
O	1.7178590000000000	4.4362290000000000	−0.7628210000000000
O	0.0877810000000000	2.5574960000000000	0.8244500000000000
O	2.7325550000000001	3.2269809999999999	1.9570680000000000

$La_2Zr_{14}O_{31}$ - D-Sym, $E_{tot} = -1\,916\,942.485\,870\,61\,\mathrm{eV}$, $\Delta E_{tot} = 14\,\mathrm{meV}$

La	11.8981890000000003	13.5206619999999997	8.5258959999999995
Zr	11.1127420000000008	13.1387850000000004	15.4475990000000003
Zr	13.6502719999999993	10.8278680000000005	9.9490409999999994
Zr	14.8808520000000009	12.1725809999999992	14.7608329999999999
Zr	14.5994089999999996	14.9481660000000005	10.5180260000000008
Zr	13.4801009999999994	15.1903559999999995	15.8047470000000008
Zr	9.2511890000000001	10.0010870000000001	12.9723950000000006
Zr	10.2192550000000004	15.1936760000000000	11.4639710000000008
La	15.1255649999999999	9.1631859999999996	12.9574529999999992
Zr	10.3653899999999997	10.6859260000000003	9.9950229999999998
Zr	12.0988690000000005	10.3014469999999996	14.7466390000000001
Zr	8.7858660000000004	12.9175059999999995	13.1499340000000000
Zr	11.9630799999999997	8.5682729999999996	11.9186490000000003
Zr	12.6359770000000005	12.8966689999999993	12.3376800000000006
Zr	12.9372319999999998	16.3825889999999994	12.9327769999999997
Zr	15.9780400000000000	12.3756959999999996	11.7263269999999995
O	9.9152319999999996	8.9061500000000002	11.3246629999999993
O	10.7099130000000002	13.5423179999999999	12.5759519999999991
O	11.3767139999999998	11.2929370000000002	16.2807420000000000
O	10.4552980000000009	14.8432650000000006	9.5925419999999999
O	15.5156259999999993	10.4497490000000006	10.9748230000000007
O	14.1818299999999997	12.7202470000000005	10.7786860000000004
O	14.9142519999999994	13.7613269999999996	15.9412719999999997
O	14.4396649999999998	16.6058149999999998	11.6573930000000008
O	7.7795649999999998	11.1268340000000006	13.5817440000000005
O	9.2703019999999992	13.5484919999999995	14.9978639999999999
O	12.1585439999999991	9.3771489999999993	10.0119659999999993
O	12.9908249999999992	10.2506039999999992	12.5065469999999994
O	13.7920010000000008	14.9136170000000003	8.8102900000000002
O	12.5362550000000006	14.6302249999999994	14.1480510000000006
O	14.6989009999999993	13.2717860000000005	13.0708739999999999
O	11.0226839999999999	8.7866710000000001	13.7990999999999993
O	10.2514950000000002	11.6641919999999999	8.3996890000000004
O	10.4923199999999994	11.3339350000000003	13.7904000000000000
O	11.1070139999999995	16.7989509999999989	12.3405269999999998
O	13.6541650000000008	7.6265130000000001	12.2751880000000000
O	13.5208899999999996	11.5832599999999992	8.2751149999999996
O	12.8133440000000007	12.2194789999999998	14.3565660000000008
O	13.6121140000000000	16.8831820000000015	14.8106349999999996
O	9.2166580000000007	11.4483779999999999	11.4749770000000009
O	8.4076280000000008	14.5639140000000005	12.0956639999999993
O	11.8747579999999999	11.9547430000000006	10.5904919999999994
O	14.2044069999999998	10.1412870000000002	14.9882310000000007
O	12.6969799999999999	14.7342910000000007	11.4980279999999997
O	11.9036349999999995	14.4457769999999996	16.7332819999999991
O	16.3496810000000004	11.3801109999999994	13.5683589999999992
O	16.4667910000000006	14.0309469999999994	10.8446669999999994

Rh/$Zr_{16}O_{32}$ - D-pGMC, $E_{tot} = -1\,777\,772.573\,107\,88$ eV, $\Delta E_{tot} = 0$ meV

Rh	−3.9478589999999998	2.7034029999999998	−1.2477769999999999
Zr	1.1654439999999999	−1.3258460000000001	−2.6601360000000001
O	−0.5602200000000001	−1.4507310000000000	−3.7166980000000001
O	0.4805460000000000	−1.5247990000000000	−0.7217880000000000
Zr	−0.0360630000000000	−2.5563570000000002	4.7645869999999997
O	−1.8765660000000000	−2.0973280000000001	4.2207689999999998
O	1.5551040000000000	−1.3993169999999999	5.0593290000000000
Zr	0.9697080000000000	−3.8374320000000002	2.1298819999999998
O	1.1341540000000001	−4.7857529999999997	0.3800920000000000
O	0.4329410000000000	−4.3206889999999998	4.0241569999999998
Zr	−2.1845319999999999	−2.1360570000000001	−2.8717410000000001
O	−1.6693870000000000	−3.7475580000000002	−1.8445879999999999
O	−3.9231950000000002	−1.5082139999999999	−3.6498379999999999
Zr	−3.1370230000000001	0.4854570000000000	0.4861130000000000
O	−2.8326590000000000	2.1207900000000000	1.6418120000000000
O	−4.7463949999999997	0.8544320000000000	−0.8953960000000000
Zr	−1.8457170000000001	−1.8690480000000000	2.1977630000000001
O	−0.8874540000000000	−3.3021170000000000	1.1069439999999999
O	−3.5770080000000002	−1.1732050000000001	1.5171460000000001
Zr	−3.9812110000000001	0.2612920000000000	−2.7099389999999999
Zr	0.0769310000000000	−3.6375980000000001	−0.8543560000000000
O	−1.4601470000000001	1.4099429999999999	−0.4874170000000000
O	−2.6340240000000001	−0.7032510000000000	−1.5147740000000001
O	0.3146430000000000	−1.9292720000000001	2.8716020000000002
O	−2.6062419999999999	1.2642359999999999	−3.7136190000000000
Zr	−1.3505339999999999	2.1636679999999999	−2.4669099999999999
O	1.2688539999999999	−3.2649319999999999	−2.4183119999999998
O	0.1621800000000000	3.3404449999999999	−3.0242689999999999
Zr	−1.6246630000000000	3.2689889999999999	0.5806500000000000
O	−0.4455170000000000	4.6288799999999997	1.5326080000000000
O	−2.2021630000000001	3.7582439999999999	−1.3120870000000000
Zr	2.3224279999999999	−1.0974010000000001	3.1959819999999999
O	−1.1610860000000001	−0.2337960000000000	1.2981499999999999
O	2.6845150000000002	−2.8470290000000000	2.3534199999999998
Zr	3.4753509999999999	1.2326990000000000	1.4126019999999999
O	1.6664779999999999	0.1744550000000000	1.6620299999999999
O	3.8974750000000000	0.1188540000000000	2.9857209999999998
Zr	3.9802119999999999	0.7568710000000000	−1.7565850000000001
Zr	0.3365720000000000	0.4018160000000000	0.0285010000000000
Zr	1.6756460000000000	2.8569079999999998	−1.8703040000000000
Zr	1.4414320000000000	4.1764400000000004	1.0311110000000001
O	4.8976769999999998	0.8654460000000000	0.0120030000000000
O	3.1064940000000001	−0.7414660000000000	−2.6908550000000000
O	3.4294419999999999	2.3823460000000001	−2.7049669999999999
O	2.4158689999999998	1.1704810000000001	−0.5652330000000000
O	0.4099470000000000	0.8618830000000000	−2.0996239999999999
O	2.0632269999999999	4.5122869999999997	−0.7765490000000000
O	0.4717180000000000	2.6374610000000001	0.1801700000000000
O	2.8546760000000000	3.0814699999999999	1.9006190000000001

Rh/$Zr_{16}O_{32}$ - D-Sym, $E_{tot} = -1\,777\,771.544\,768\,03\,\text{eV}$, $\Delta E_{tot} = 21\,\text{meV}$

Rh	−3.3828480000000001	−1.7896630000000000	2.5244059999999999
O	−3.1704260000000000	−2.6195819999999999	−2.1033430000000002
O	−1.4264589999999999	1.8841030000000001	−0.1124370000000000
O	−1.8784500000000000	−1.3673630000000001	3.8371149999999998
O	−2.2909709999999999	3.1687059999999998	−2.8546589999999998
O	1.6188389999999999	−3.3422299999999998	−2.0805020000000001
O	1.5935180000000000	−0.1998530000000000	−1.4341740000000001
O	2.7400460000000000	1.0885670000000001	4.3595990000000002
O	0.9762950000000000	2.7977129999999999	−1.5852790000000001
O	−4.1018530000000002	−1.2571460000000001	0.7203349999999999
O	−2.9973559999999999	1.6296459999999999	2.4463420000000000
O	−0.6132630000000000	−2.0270030000000001	−4.0269700000000004
O	0.9004370000000000	−1.7550360000000000	0.7748790000000000
O	0.5343440000000000	2.2265980000000001	−4.4391870000000004
O	1.1086620000000000	1.2818790000000000	0.6481280000000000
O	3.7471260000000002	−2.1244820000000000	3.3140139999999998
O	3.6448529999999999	1.9193039999999999	1.3094730000000001
O	−1.1295400000000000	−3.5221220000000000	0.7788700000000000
O	−2.5367020000000000	0.3064720000000000	−4.3020090000000000
O	−1.1339129999999999	−0.2746770000000000	1.5126040000000001
O	−1.3225450000000001	4.5109190000000003	−0.1022220000000000
O	2.2831690000000000	−3.9123839999999999	0.7896130000000000
O	2.0513330000000001	−0.3464490000000000	−3.9990480000000002
O	2.3050009999999999	−0.2851770000000000	2.2211609999999999
O	1.2812640000000000	3.6248410000000000	1.8528450000000001
O	−2.3608389999999999	−0.3414430000000000	−0.9244090000000000
O	−4.1297740000000003	2.2205240000000002	−0.2231610000000000
O	−0.5726920000000000	−1.9220010000000001	−1.3250329999999999
O	0.8125900000000000	−2.0543559999999998	3.7290320000000001
O	−0.4989800000000000	0.4700270000000000	−2.5992799999999998
O	0.1074690000000000	1.3667140000000000	3.4835360000000000
O	3.9778210000000001	−1.0711489999999999	0.1150500000000000
O	3.5084080000000002	1.5781890000000001	−1.9714600000000000
Zr	−1.2827660000000001	1.8191090000000001	−3.9032010000000001
Zr	−1.6106270000000000	0.6199440000000001	3.3798020000000002
Zr	1.0418620000000001	−1.6614279999999999	−2.9835790000000002
Zr	2.4513650000000000	−0.8416600000000000	4.0784050000000001
Zr	1.7825260000000001	1.4005559999999999	−2.9827849999999998
Zr	2.0669940000000002	1.8799690000000000	2.5670370000000000
Zr	−2.6165140000000000	−2.3074840000000001	−0.2829360000000000
Zr	−2.6145109999999998	3.3367830000000001	−0.9424030000000000
Zr	3.0175860000000001	−2.1159260000000000	1.4416040000000001
Zr	−2.0291530000000000	−1.1467790000000000	−3.0392809999999999
Zr	−0.6084410000000000	−2.1478100000000002	2.3654760000000001
Zr	−3.2838810000000000	0.7090850000000000	0.6930250000000000
Zr	0.7400510000000000	−3.4778349999999998	−0.3163120000000000
Zr	−0.1798280000000000	0.0453730000000000	−0.4014030000000000
Zr	0.2906610000000000	3.2659419999999999	0.1911180000000000
Zr	3.1901069999999998	0.7600749999999999	−0.1983930000000000

$Rh/La_2Zr_{14}O_{31}$ - D-pGMC, $E_{tot} = -2\,048\,557.854\,876\,17\,\text{eV}$, $\Delta E_{tot} = 9\,\text{meV}$

Rh	1.6314949999999999	0.0372290000000000	−3.3194659999999998
La	2.2094770000000001	−2.5574659999999998	−1.3204880000000001
Zr	0.1591230000000000	−3.0483730000000002	4.1105799999999997
La	1.6994030000000000	−4.8357669999999997	1.5552189999999999
Zr	−1.2442219999999999	−2.1406120000000000	−3.0164190000000000
Zr	−3.4652720000000001	0.7301090000000000	1.1091480000000000
Zr	−2.3490940000000000	−1.7495229999999999	2.6228440000000002
Zr	−3.1876509999999998	0.1137230000000000	−2.0257849999999999
Zr	−0.9928470000000000	−2.8483200000000002	0.0016920000000000
Zr	−1.6286240000000001	2.7938200000000002	−2.4266950000000000
Zr	−1.8958349999999999	3.6044960000000001	0.6753820000000000
Zr	1.9526319999999999	−1.2849950000000001	2.1424699999999999
Zr	3.0708839999999999	1.4698439999999999	1.2083420000000000
Zr	3.7713369999999999	0.6330710000000001	−1.7315600000000000
Zr	−0.0997800000000000	0.4883900000000000	−0.0767460000000000
Zr	1.4320310000000001	2.7010339999999999	−2.4490799999999999
Zr	1.1350750000000001	4.1688820000000000	0.7997130000000000
O	0.7107530000000000	−1.8319040000000000	−3.1934079999999998
O	0.7087120000000000	−1.4457180000000001	−0.0370280000000000
O	−1.7172980000000000	−2.4268709999999998	4.3977050000000002
O	1.6510030000000000	−1.7803410000000000	4.0624300000000000
O	0.4774140000000000	−4.2620920000000000	−0.1271730000000000
O	0.8243790000000000	−4.8221769999999999	3.5880670000000001
O	−1.4888079999999999	−3.6167560000000001	−1.8210059999999999
O	−2.9438810000000002	−1.2391870000000000	−3.4897070000000001
O	−3.3119710000000002	2.6285080000000001	1.6896880000000001
O	−4.4489010000000002	0.0626110000000000	−0.5108680000000000
O	−2.5702889999999998	−3.0701450000000001	1.1717569999999999
O	−3.9044140000000001	−0.5381650000000000	2.5881630000000002
O	−2.1504170000000000	1.4878240000000000	−0.5850350000000000
O	−1.4678460000000000	−1.0146400000000000	−1.3051960000000000
O	−0.1189040000000000	−2.5499600000000000	2.1945150000000000
O	−3.0662799999999999	1.7189700000000001	−3.1928670000000001
O	−0.0696260000000000	3.5235319999999999	−3.4496560000000001
O	−0.6114780000000000	4.9769280000000000	1.3773700000000000
O	−2.2322050000000000	4.1950149999999997	−1.1578379999999999
O	−1.6101859999999999	−0.2514390000000000	1.3531120000000001
O	2.7281380000000000	−2.9504540000000001	1.1395500000000001
O	1.1409080000000000	0.5139790000000000	1.6297960000000000
O	3.5812439999999999	−0.1711710000000000	2.1913239999999998
O	4.5557930000000004	1.5288349999999999	−0.1423480000000000
O	4.0006610000000000	−1.2914150000000000	−1.5605210000000000
O	2.9985860000000000	1.7488740000000000	−3.3229090000000001
O	2.0703190000000000	1.1947580000000000	−0.6530670000000000
O	−0.0915440000000000	1.3594710000000001	−2.0440390000000002
O	1.7427440000000001	4.1492800000000001	−1.0619220000000000
O	−0.1081140000000000	2.6010080000000002	0.5359159999999999
O	2.5233699999999999	3.2973040000000000	1.8760390000000000

Rh/$La_2Zr_{14}O_{31}$ - D-pGMC, $E_{tot} = -2\,048\,558.307\,770\,50$ eV, $\Delta E_{tot} = 0$ meV

Rh	−2.5712090000000001	0.6426950000000000	−3.8447130000000000
La	0.0219110000000000	1.2286740000000000	−3.8173469999999998
Zr	−1.1807589999999999	0.5767900000000000	3.0856210000000002
Zr	1.3264530000000001	−1.6556139999999999	−2.4713180000000001
Zr	2.5455359999999998	−0.3962720000000000	2.3583680000000000
Zr	2.4690140000000000	2.5182199999999999	−1.5993839999999999
Zr	1.1725760000000001	2.6130849999999999	3.5200309999999999
Zr	−3.0981980000000000	−2.4581149999999998	0.5554590000000000
Zr	−2.0791390000000001	2.6881889999999999	−0.9265080000000000
La	2.7629929999999998	−3.4009239999999998	0.5124670000000000
Zr	−2.0256669999999999	−1.7193240000000001	−2.4288780000000001
Zr	−0.2415610000000000	−2.2425899999999999	2.3126060000000002
Zr	−3.5135209999999999	0.4443340000000000	0.7920920000000000
Zr	−0.4002010000000000	−3.8914680000000001	−0.5762310000000000
Zr	0.3404140000000000	0.3824110000000000	−0.0870860000000000
Zr	0.5890770000000000	3.8600829999999999	0.6990900000000000
Zr	3.6928000000000001	−0.1919960000000000	−0.6503110000000000
O	−2.4338869999999999	−3.4784009999999999	−1.1484970000000001
O	−1.5730999999999999	1.0505100000000001	0.1780800000000000
O	−0.9164530000000000	−1.2835620000000001	3.8863170000000000
O	−1.9119510000000000	2.3584580000000002	−2.8565309999999999
O	3.1768160000000001	−2.0927370000000001	−1.4511120000000000
O	1.9390989999999999	0.2490000000000000	−1.6465799999999999
O	2.6009560000000000	1.1656810000000000	3.5714769999999998
O	2.2143079999999999	4.1070669999999998	−0.4021030000000000
O	−4.5440690000000004	−1.3181309999999999	1.2205320000000000
O	−3.0033759999999998	1.0609100000000000	2.6371820000000001
O	−0.1784240000000000	−3.0693180000000000	−2.4620069999999998
O	0.6568760000000000	−2.2452830000000001	0.0546190000000000
O	1.8191310000000001	2.6889059999999998	−3.3613940000000002
O	0.2303780000000000	2.0921530000000002	1.8435280000000001
O	2.3940030000000001	0.7269090000000000	0.6745030000000000
O	−1.3391820000000001	−3.7092760000000000	1.3188480000000000
O	−2.9383849999999998	−1.3070379999999999	−4.0353510000000004
O	−1.8469439999999999	−1.1583150000000000	1.4289689999999999
O	−1.2162610000000000	4.2675190000000001	−0.0060690000000000
O	1.2672470000000000	−4.8793080000000000	−0.2092280000000000
O	1.2174060000000000	−0.8500380000000000	−4.1501380000000001
O	0.4910730000000000	−0.3266980000000000	1.9373520000000000
O	1.2202530000000000	4.3473649999999999	2.5910000000000002
O	−3.1047370000000001	−0.9830590000000000	−0.8639380000000000
O	−3.8692510000000002	2.1136520000000001	−0.2634330000000000
O	−0.3836290000000000	−0.5015579999999999	−1.8464620000000000
O	1.8593090000000001	−2.4289580000000002	2.5487690000000001
O	0.4450430000000000	2.2594029999999998	−0.8562480000000000
O	−0.3862130000000000	1.8338770000000000	4.4255420000000001
O	4.0154699999999997	−1.1981210000000000	1.1660550000000001
O	4.2879839999999998	1.5102100000000001	−1.3576410000000001
